# NRF2 as a Therapeutic Target in Dermatological Disorders: Mechanisms and Molecules

**DOI:** 10.3390/ph19030497

**Published:** 2026-03-17

**Authors:** Ismael Khiar-Fernández, Nora Khiar-Fernández, José-Juan Pereyra-Rodríguez, Inmaculada Fernández

**Affiliations:** 1 Dermatology Department, Hospital Universitario Virgen del Rocío, 41013 Sevilla, Spain; ismkhifer@alum.us.es (I.K.-F.); jpereyra@us.es (J.-J.P.-R.); 2 Medicine Department, Universidad de Sevilla, 41009 Sevilla, Spain; 3 Departamento de Química Orgánica, Facultad de Química, Universidad Complutense de Madrid, 28040 Madrid, Spain; nkhiar@ucm.es; 4 Departamento de Química Orgánica y Farmacéutica, Facultad de Farmacia, Universidad de Sevilla, 41012 Sevilla, Spain

**Keywords:** KEAP1-NRF2 pathway, dermatological disorders, skin diseases, antioxidant response element (ARE), oxidative stress

## Abstract

The nuclear factor erythroid 2–related factor 2 (NRF2) is a master transcription factor that orchestrates cellular defense against oxidative and electrophilic stress. Dysregulation of the KEAP1–NRF2–ARE pathway has been implicated in several dermatological disorders, including vitiligo, psoriasis, atopic dermatitis, photoaging, and radiation dermatitis. This review summarizes recent advances in the understanding of NRF2 activation mechanisms and highlights pharmacological and natural compounds with potential dermatological applications. A comprehensive analysis of natural, semisynthetic, and synthetic NRF2 modulators is provided, describing their chemical structures, synthetic approaches, mechanisms of action, preclinical and clinical evidence, and therapeutic relevance for skin disorders. Multiple classes of NRF2 activators, including isothiocyanates such as sulforaphane, triterpenoids such as omaveloxolone, flavonoids including baicalein and apigenin, alkaloids such as berberine, glycosides like afzelin and paeoniflorin, stilbenoids such as tapinarof, and α,β-unsaturated fumaric acid esters such as dimethyl fumarate, have demonstrated antioxidant, anti-inflammatory, and cytoprotective effects in keratinocytes and melanocytes. Some of these agents, particularly dimethyl fumarate and tapinarof, have advanced to clinical development or commercialization, whereas others remain at the preclinical stage but show encouraging results in animal models and cell culture systems. Overall, pharmacological activation of NRF2 represents a promising therapeutic strategy to counteract oxidative stress–driven skin damage and inflammation; however, continued translational and clinical research is required to optimize formulations, dosing regimens, and safety profiles for integration into dermatological practice.

## 1. Introduction

The transcription factor nuclear factor erythroid 2-related factor 2 (NRF2) has emerged as a pivotal regulator of cellular antioxidant and detoxification responses. Increasing evidence links NRF2 dysregulation to the pathogenesis of various dermatological disorders, including inflammatory conditions, pigmentary disorders, and photoaging [1,2,3,4]. Accordingly, the NRF2 signaling pathway is now recognized as a promising therapeutic target in the management of skin diseases [5,6,7]. This review examines the activation mechanisms of NRF2, associated skin diseases, and candidate molecules developed to modulate this pathway.

**Figure 1:**
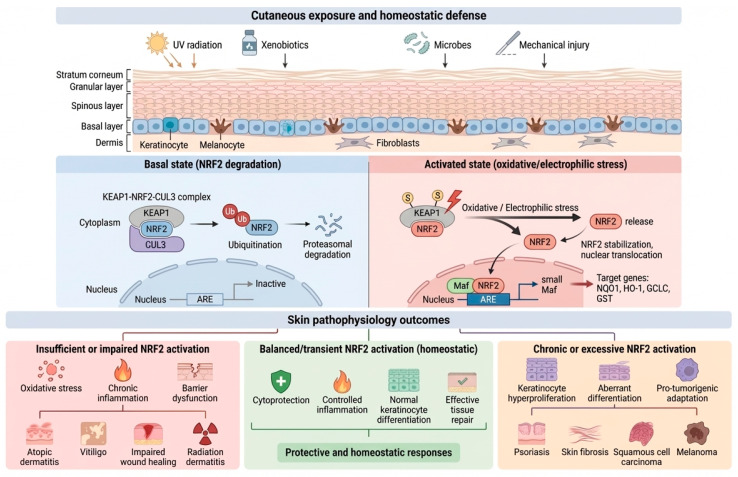
NRF2 signaling in skin: KEAP1-mediated degradation under basal conditions versus stress-induced NRF2 stabilization, nuclear translocation, and ARE-driven gene expression, reflecting insufficient, balanced, or excessive activation in cutaneous disease.

Beyond its role as a generic antioxidant switch, the KEAP1–NRF2–ARE axis is increasingly regarded as a regulatory hub that integrates the cutaneous exposome with barrier biology and immune signaling [5,6]. In the epidermis, NRF2 activity influences keratinocyte differentiation, redox buffering, xenobiotic metabolism, and stress-adaptive programs that shape inflammatory tone and tissue repair [6,7]. This translational interest is accompanied by an important nuance: while moderate and time-limited NRF2 activation is typically cytoprotective, sustained pathway hyperactivation in chronically inflamed environments may contribute to maladaptive outcomes such as aberrant differentiation or hyperproliferation [8] (Figure 1).

NRF2 activation, therefore, requires an appropriate magnitude, duration, and tissue localization of pathway engagement, often achieved through topical delivery and chemically defined small molecules. In this context, we first outline the principal molecular mechanisms governing NRF2 regulation and then critically review candidate NRF2 modulators for skin disorders, emphasizing their chemical features, available synthetic information, and the strength of preclinical and clinical evidence.

## 2. NRF2: Structure and Activation Pathways

### 2.1. NRF2 Protein Structure

**Figure 2:**
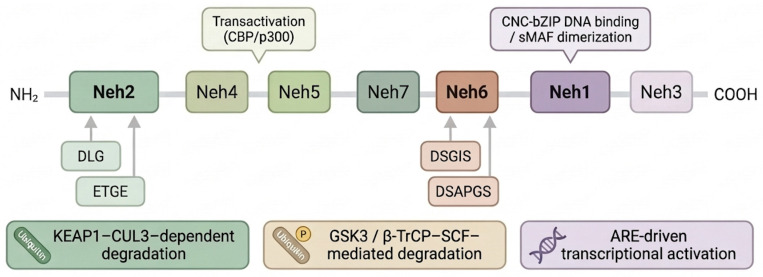
Schematic domain organization of human NRF2 (*NFE2L2*). Neh2 contains *KEAP1*-binding motifs (DLG and ETGE), whereas Neh6 contains β-TrCP degron motifs (DSGIS, DSAPGS). Neh4/5 mediate transactivation, and Neh1 enables CNC-bZIP DNA binding.

NRF2 (nuclear factor erythroid 2–related factor 2), encoded by the *NFE2L2 gene*, is a stress-responsive transcription factor that orchestrates cellular defense programs against oxidative and electrophilic insults. NRF2 comprises seven NRF2-ECH homology domains (Neh1–Neh7) that coordinate its DNA binding, transcriptional activity, and regulated turnover (Figure 2).

The Neh2 domain harbors two KEAP1-binding motifs, ETGE (high affinity) and DLG (lower affinity), which enable KEAP1-dependent ubiquitination and proteasomal degradation under basal conditions [2]. The Neh1 domain contains the CNC-bZIP region required for heterodimerization with small Maf (sMAF) proteins and binding to antioxidant response elements (AREs). Transactivation is mediated largely by Neh4 and Neh5, which recruit co-activators such as CBP/p300, while Neh3 contributes to C-terminal transcriptional activation. The Neh6 domain provides a KEAP1-independent degradation route via degron motifs (including DSGIS and DSAPGS) that can be recognized by β-TrCP within the SCF (CUL1-based) E3 ligase complex. Finally, Neh7 has been described as a negative regulatory module through interactions with RXRα in specific cellular contexts [1,9].

### 2.2. Canonical Activation Pathway (KEAP1–CUL3 Axis)

Under homeostatic conditions, KEAP1 functions as a substrate adaptor for a *CUL3*-dependent E3 ubiquitin ligase complex, thereby maintaining low levels of NRF2 through continuous ubiquitin–proteasome mediated turnover [3,4] (Figure 1, “*Basal state*”). During oxidative or electrophilic stress, modification of key KEAP1 cysteine residues can impair the ability of the KEAP1–CUL3 ligase system to ubiquitinate NRF2 [10]. Consequently, NRF2 becomes stabilized, accumulates, and translocates to the nucleus, where it binds AREs and drives a broad cytoprotective transcriptional program involved in antioxidant defense, detoxification, and metabolic adaptation (Figure 1, “*Activated state*”). This KEAP1-centered mechanism accounts for the rapid and typically transient activation of NRF2 in response to redox imbalance [1].

### 2.3. Non-Canonical Activation Pathway (P62/Sqstm1–Autophagy Axis)

Beyond canonical KEAP1 cysteine sensing, NRF2 can also be activated through non-canonical mechanisms that alter KEAP1 availability. A major route involves p62/*SQSTM1***,** an autophagy adaptor protein. Upon phosphorylation (notably at Ser349 within its KEAP1-interacting region), p62 binds KEAP1 and promotes its sequestration and/or turnover, thereby reducing the pool of functional KEAP1 and allowing NRF2 to accumulate and activate nuclear ARE-driven transcription. This pathway is particularly relevant in settings where p62 accumulates due to impaired or overloaded autophagy [11].

### 2.4. Kinase-Mediated Regulation and KEAP1-Independent Turnover

NRF2 signaling is further tuned by kinase-dependent events that influence NRF2 release, nuclear localization, and turnover. For example, PKC-dependent phosphorylation of NRF2 (e.g., at Ser40) has been associated with reduced KEAP1-mediated restraint in some experimental settings. In parallel, the GSK-3β/β-TrCP pathway provides a KEAP1-independent mechanism for limiting NRF2: GSK-3β promotes phosphorylation-dependent recognition of NRF2 Neh6 degrons by β-TrCP, facilitating SCF-mediated ubiquitination and degradation. In addition, GSK-3β–Fyn signaling has been linked to NRF2 nuclear export in certain contexts, adding a further layer of spatial control over NRF2 activity. Collectively, these pathways enable context-dependent fine-tuning of NRF2 signaling amplitude and duration beyond the KEAP1–CUL3 system [3,12].

### 2.5. Transcriptional/Epigenetic Modulation and Pathway Feedback

In addition to post-translational regulation, NRF2 signaling output can be influenced by changes in *NFE2L2* and *KEAP1* expression, including transcriptional and epigenetic mechanisms (e.g., promoter methylation and chromatin-associated regulation) that have been reported in chronic disease contexts. Importantly, NRF2 also participates in feedback control of the pathway by regulating components of its own regulatory network, including KEAP1, thereby contributing to the restoration of homeostasis once the stress response subsides. Dysregulated or persistent NRF2 activity may be associated with maladaptive consequences in specific pathological settings, emphasizing the importance of controlled and context-appropriate pathway engagement [1,4,13,14].

## 3. NRF2 and Skin Pathophysiology

The skin is constantly exposed to ultraviolet (UV) radiation, xenobiotics, microbes, and mechanical injury, and therefore relies on inducible cytoprotective systems to maintain homeostasis. Among these, the KEAP1–NRF2–ARE axis represents a central regulator of cutaneous defense. Under basal conditions, KEAP1 targets NRF2 for ubiquitin–proteasome degradation, thereby maintaining low basal NRF2 activity. When activated through any of the mechanisms described above, NRF2 escapes degradation, accumulates in the nucleus, and binds antioxidant response elements (AREs), thereby initiating a broad cytoprotective transcriptional program. Collectively, available data indicate that NRF2 activity contributes to keratinocyte viability, epidermal barrier integrity, and immune homeostasis [5,6,7] (Figure 1). Insufficient pathway activation may render the skin more susceptible to oxidative injury and chronic inflammation; by contrast, sustained or excessive NRF2 signaling may favor hyperproliferation, aberrant differentiation or even oncogenic progression. Accordingly, dysregulation of KEAP1–NRF2–ARE signaling has been implicated in psoriasis, atopic and allergic dermatitis, vitiligo, photodamage and photoaging, impaired wound healing, skin fibrosis, and both melanoma and non-melanoma skin cancer [5,6,7].

In the following subsections, we summarize how NRF2 contributes to the pathophysiology of major dermatological entities, leaving a detailed discussion of pharmacological NRF2 activators for the subsequent section.

### 3.1. Psoriasis

Psoriasis is a chronic immune-mediated dermatosis characterized by keratinocyte hyperproliferation, altered differentiation, and IL-23/IL-17-driven inflammation. Elevated oxidative stress and reduced antioxidant capacity have been consistently reported in affected patients, pointing to a relevant contribution of redox imbalance to disease pathogenesis [6].

Accumulating experimental and translational evidence suggests that NRF2 exerts context-dependent effects in psoriasis. Moderate activation of NRF2 enhances antioxidant defenses and suppresses NF-κB-dependent pro-inflammatory cytokines, which can attenuate the IL-23/IL-17 axis and reduce the severity of experimental psoriasiform dermatitis [6,15]. At the same time, NRF2 also induces so-called “stress keratins” such as K6, K16, and K17 as well as other genes that promote keratinocyte proliferation and acanthosis, features that are typical of psoriatic plaques [6]. Consistently, mouse models with epidermal NRF2 overactivity show psoriasiform lesions with strong expression of these keratins, illustrating how a pathway that is fundamentally cytoprotective can contribute to pathological hyperplasia under chronic stimulation [6]. Taken together, available data support the hypothesis that transient or moderate NRF2 activation is protective, whereas sustained activation within a chronically inflamed environment may promote epidermal hyperproliferation and plaque persistence [6,7]. This nuance is highly relevant for therapeutic strategies that aim to modulate NRF2 in psoriasis.

### 3.2. Atopic and Allergic Dermatitis

Atopic dermatitis (AD) arises from a combination of genetic barrier defects, type-2-skewed immunity, and heightened susceptibility to environmental triggers. Oxidative stress markers are elevated in AD, and recent proteomic and transcriptomic studies suggest that epidermal NRF2 activity is reduced in lesional skin compared with healthy controls [16,17].

NRF2 intersects with AD pathophysiology at multiple levels. First, it contributes to the regulation of cornified envelope proteins such as filaggrin, loricrin, and involucrin. These proteins are influenced not only by cytokines (IL-4, IL-13, IL-17A, IL-22) but also by AhR–NRF2 crosstalk; adequate NRF2 activity appears to support terminal differentiation and barrier formation, whereas dysregulation may exacerbate the filaggrin–loricrin–involucrin deficiency characteristic of AD [16]. Second, by strengthening antioxidant defenses and negatively regulating NF-κB and JAK–STAT pathways, NRF2 activation has been shown in experimental models to limit type-2 cytokine production and pruritogenic mediators, thereby attenuating AD-like inflammation [6,17].

The KEAP1–NRF2 system also functions as a sensor of electrophilic allergens and irritants. Contact sensitizers rapidly activate NRF2 in keratinocytes and dendritic cells; this early response facilitates adaptation to oxidative stress but also influences antigen presentation and the development of contact hypersensitivity [17]. Depending on the magnitude and duration of activation, NRF2 may either promote the resolution of inflammation or contribute to the persistence of chronic eczematous disease [6,17].

### 3.3. Vitiligo and Melanocyte Survival

Vitiligo is a depigmenting disorder in which melanocytes are lost through the combined effects of oxidative stress, metabolic disturbance and autoimmune attack. Lesional melanocytes and keratinocytes exhibit increased ROS, impaired mitochondrial function, and reduced activity of antioxidant enzymes such as catalase [18]. Oxidative stress in this setting promotes the release of danger signals, alters melanocyte antigens, and facilitates cytotoxic T-cell responses, thereby linking redox imbalance to autoimmunity [18].

NRF2 functions as a major transcriptional regulator of antioxidant responses in melanocytes in the face of this oxidative burden. When activated, NRF2 induces enzymes such as HO-1, NQO1, and GCLC that restore redox balance and support mitochondrial homeostasis, favoring melanocyte survival [18]. Genetic and metabolomic studies suggest that variants in *NFE2L2* and several NRF2-regulated antioxidant genes may influence vitiligo susceptibility and disease course [19].

NRF2 also interacts with pathways involved in melanogenesis and melanocyte stem-cell maintenance, including Wnt/β-catenin signaling [20]. Reduced NRF2 activity could therefore contribute not only to melanocyte loss but also to impaired repigmentation. Conversely, in melanoma, constitutive NRF2 activation supports tumor survival under oxidative and therapeutic stress, highlighting the need for disease-specific NRF2 modulation [7].

### 3.4. Photoaging, Photodamage and Radiation Dermatitis

The skin is uniquely exposed to solar UV and visible radiation, which generate ROS, DNA lesions, and chronic low-grade inflammation. The KEAP1–NRF2 axis coordinates a substantial component of the intrinsic defense against these insults. In preclinical models, enhancement of NRF2 activity in the epidermis has been shown to increase antioxidant and phase-II detoxifying enzymes and to reduce UV-induced oxidative damage, inflammation, and apoptosis in keratinocytes and fibroblasts [5,20,21,22]. These NRF2-dependent responses translate into reduced photodamage and delayed photocarcinogenesis in hairless mice repeatedly exposed to solar-simulated radiation [5,21,22,23].

NRF2 has also been linked to premature and chronological skin aging. Work in Hutchinson–Gilford progeria syndrome demonstrated that progerin can sequester NRF2 at the nuclear lamina, thereby impairing its transcriptional activity and exacerbating oxidative stress; related mechanisms have been proposed to contribute to intrinsic photoaging in chronically sun-exposed skin [24].

Ionizing radiation used in cancer therapy produces an even more intense oxidative burden. Experimental data suggest that intact NRF2 signaling mitigates dermal and epidermal damage and reduces the severity of radiation-induced dermatitis, whereas insufficient NRF2 responses are associated with more severe acute lesions and impaired recovery [25,26]. Together, these observations support a central role for NRF2 in protecting the skin against both solar and therapeutic radiation, while also highlighting the need to avoid excessive pathway activation.

### 3.5. Wound Healing and Diabetic Ulcers

Cutaneous wound repair requires a tightly coordinated sequence of hemostatic, inflammatory, proliferative, and remodeling phases, all of which are sensitive to the redox environment. Experimental data indicate that NRF2 contributes to this coordination by limiting excessive oxidative stress, supporting keratinocyte and fibroblast survival, and modulating inflammatory gene expression [27].

In diabetic settings, chronic hyperglycemia and microangiopathy lead to sustained oxidative stress and a relative inadequacy of NRF2 activation. Experimental work shows that suppression of NRF2 signaling delays wound closure, prolongs inflammation, and impairs granulation tissue formation, whereas restoration of NRF2 activity normalizes antioxidant responses, improves re-epithelialization, and enhances collagen deposition [28,29,30]. These findings place NRF2 at the center of the impaired healing characteristic of diabetic foot ulcers and other chronic wounds.

### 3.6. Skin Fibrosis and Sclerosing Dermatoses

Fibrosing skin disorders, including systemic sclerosis, chronic radiation fibrosis, and hypertrophic scars, are driven by persistent myofibroblast activation and excessive extracellular matrix deposition. Oxidative stress and TGF-β signaling are important contributors to this process [31].

Although data are more limited than in other fields, available experimental evidence suggests that NRF2 may exert anti-fibrotic effects. Reduced NRF2 activity in keratinocytes and dermal fibroblasts is associated with increased ROS, enhanced expression of pro-inflammatory chemokines, and exaggerated matrix production [31]. In animal models, restoration of NRF2 signaling can attenuate dermal thickening and collagen accumulation, indicating that adequate NRF2 activity in the epidermis and dermis helps to restrain pro-fibrotic cascades [32]. Further work is needed to clarify how these findings translate to human conditions such as systemic sclerosis or chronic graft-versus-host disease.

### 3.7. Skin Cancer

The relationship between NRF2 and cutaneous carcinogenesis is highly stage dependent. During tumor initiation, NRF2 appears to behave predominantly as a tumor suppressor: by enhancing antioxidant capacity and detoxification, it reduces ROS-induced DNA damage and mutagenesis in keratinocytes [5,7]. In several chemical and UV-induced carcinogenesis models, loss of *Nrf2* is associated with increased numbers and size of papillomas and squamous cell carcinomas, whereas genetic enhancement of NRF2 activity reduces tumor burden [33,34]. Constitutive NRF2 activation in established tumors promotes metabolic reprogramming that supports redox homeostasis and anabolic growth. NRF2 transcriptionally regulates enzymes involved in NADPH regeneration (e.g., glucose-6-phosphate dehydrogenase, malic enzyme 1) and glutathione biosynthesis (e.g., *GCLC*, *GCLM*), thereby enhancing intracellular reducing capacity and resistance to oxidative stress. This metabolic rewiring contributes to tumor survival, chemoresistance, and adaptation to hostile microenvironments [35,36].

Once tumors are established, however, many acquire somatic mutations in *NFE2L2* or in components of the *KEAP1*–*CUL3* E3 ligase complex, resulting in chronic NRF2 activation. This constitutive activity supports anabolic metabolism, protects malignant cells from therapy-induced stress, and contributes to immune evasion [37]. Recent work in mouse skin has shown that different modes of NRF2 gain-of-function (e.g., activating mutations versus *Keap1* loss) can drive distinct squamous cell fates and influence the spectrum of cooperating oncogenic mutations [35]. Activating mutations in *NFE2L2* typically disrupt KEAP1-binding motifs within the Neh2 domain, leading to impaired ubiquitination and constitutive nuclear accumulation of NRF2. In contrast, loss-of-function alterations in *KEAP1* reduce its ability to target NRF2 for proteasomal degradation. Although both alterations result in persistent NRF2 activation, differences in mutational context and co-occurring genomic events may influence tumor phenotype and metabolic dependency [37].

A similar pattern is emerging in melanoma and other malignancies, where high NRF2 activity correlates with treatment resistance and poor prognosis. NRF2 can thus be viewed as a paradoxical regulator during early carcinogenesis that may confer a selective advantage when locked in a hyperactive state in established tumors [7,35,36]. From a translational perspective, these observations underscore the importance of exposure control and duration when considering NRF2-activating therapies. Although topical application generally results in localized and transient pathway engagement, the long-term safety of sustained NRF2 activation in chronically UV-exposed or premalignant skin warrants careful evaluation.

### 3.8. Other Inflammatory Dermatoses and Emerging Connections

Oxidative stress and redox imbalance have been described in several other inflammatory dermatoses. Increased markers of oxidative damage have been reported in lichen planus, chronic urticaria, and hidradenitis suppurativa, among others [38,39,40]. Although direct data on KEAP1–NRF2 signaling in these conditions are still scarce, it is plausible that inadequate NRF2 activation contributes to the persistent inflammation and tissue damage seen in these diseases.

NRF2 also intersects with pathways controlling keratinocyte differentiation, sebaceous gland function, and hair-follicle cycling, raising the possibility that dysregulated NRF2 contributes to disorders such as seborrheic dermatitis or certain alopecias, although this remains speculative [6,7]. As mechanistic and clinical data accumulate, it is likely that the NRF2 axis will be linked to an even broader spectrum of cutaneous pathology.

### 3.9. Conceptual Framework: Therapeutic Windows and Context-Dependent NRF2 Modulation in Dermatology

NRF2 activation in the skin cannot be considered uniformly beneficial or detrimental; rather, its biological effects are highly context-dependent and disease-specific. A translationally relevant framework requires distinguishing between insufficient, balanced, and excessive pathway activation.

Under physiological conditions, low basal NRF2 activity maintains redox homeostasis while allowing normal keratinocyte differentiation and immune surveillance [1,41]. In several dermatological disorders, including vitiligo, atopic dermatitis, chronic wounds, and radiation dermatitis, evidence suggests a relative NRF2 insufficiency or impaired stress-adaptive responses. Reduced NRF2 signaling has been associated with increased oxidative stress, mitochondrial dysfunction, and impaired barrier resilience in inflammatory skin disease [16]. In these contexts, pharmacological enhancement of NRF2 can restore antioxidant defenses, limits NF-κB–driven inflammatory amplification, and promote tissue protection [5,7]. Here, NRF2 activation functions as a mechanistically corrective intervention.

However, the magnitude and duration of activation critically determine the biological outcome. In psoriasis, transient NRF2 activation may attenuate oxidative stress and inflammatory cytokine production [6,15], whereas sustained or epidermis-restricted hyperactivation has been shown to promote stress keratin expression (K6, K16, K17), acanthosis, and psoriasiform phenotypes in experimental models [6,42]. Thus, therapeutic strategies in psoriasis likely require controlled, localized, and time-limited pathway activation.

The dual nature of NRF2 is most evident in skin carcinogenesis. During tumor initiation, NRF2 behaves predominantly as a tumor suppressor by limiting ROS-induced DNA damage and mutagenesis [7]. In contrast, established tumors frequently exploit constitutive NRF2 activation, often via *KEAP1* or *NFE2L2* mutations, to enhance metabolic reprogramming, cytoprotection, immune evasion, and therapeutic resistance [8,36,43]. In this setting, chronic pharmacological activation may be undesirable, particularly in patients with premalignant lesions or established non-melanoma skin cancer.

Beyond keratinocytes, NRF2 signaling also exhibits cell-type–specific effects in melanocytes, dermal fibroblasts, and cutaneous immune cells, further reinforcing the context-dependent nature of therapeutic modulation [44,45].

Collectively, these observations support a disease-stratified “therapeutic window” model for NRF2 modulation in dermatology, in which insufficient NRF2 activity increases oxidative vulnerability and inflammatory amplification, moderate and time-limited activation promotes cytoprotection and barrier reinforcement, and sustained or excessive activation may favor hyperproliferation, altered differentiation, or tumor support. Accordingly, NRF2-targeted strategies should be tailored to the disease context and aim for controlled pathway engagement [1,7].

The key distinctions between acute physiological activation and chronic hyperactivation across relevant cutaneous cell types are summarized in Table 1.

## 4. NRF2-Activating Compounds for Skin Disorders

A wide range of natural, semisynthetic, and synthetic compounds have been identified as modulators of the KEAP1–NRF2–ARE signaling axis, offering therapeutic potential for oxidative stress-mediated skin disorders. These molecules differ in origin, chemical structure, and their mechanisms of NRF2 activation. In the following section, we provide a detailed overview of selected NRF2-activating compounds, including their chemical structure, synthesis (where available), mechanism of action, dermatological applications, and current development status. Although some phytochemicals have shown potential in dermatology [46,47,48,49,50], this review focuses on pure bioactive compounds with rigorously defined chemical structures. However, the strength of evidence supporting direct KEAP1-dependent NRF2 activation varies among compounds, and in several cases NRF2 engagement appears secondary to broader redox modulation rather than a primary cysteine-sensing mechanism.

To facilitate interpretation, the compounds discussed below are organized by translational maturity in dermatology (from highest to lowest): (i) agents approved for dermatologic indications (tapinarof; dimethyl fumarate), (ii) approved drugs/supplements with clinical dermatology use or repurposing interest (indomethacin; simvastatin; folic acid), (iii) clinical-stage candidates evaluated in dermatologic settings (omaveloxolone; sulforaphane; molecular hydrogen), and (iv) preclinical candidates supported by skin-relevant models (bixin; 6-shogaol/thiophene analogs; paeoniflorin; baicalein; berberine; apigenin; afzelin).

### 4.1. Approved NRF2-Related Small Molecules in Dermatology

**Figure 3:**
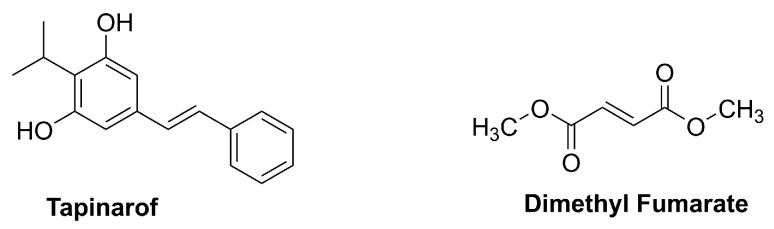
Structures of approved NRF2-related small molecules in dermatology.

This section summarizes chemically defined small molecules (Figure 3) with dermatology regulatory approval and mechanistic links to oxidative-stress pathways relevant to NRF2 signaling, representing the highest level of translational maturity for skin indications.

#### 4.1.1. Tapinarof (Vtama^®^, Benvitimod)

Tapinarof, also known as benvitimod, (*E*)-3,5-dihydroxy-4-isopropylstilbene (DHPS), is a chemically defined stilbenoid built on a *trans*-stilbene scaffold bearing a resorcinol-type phenolic motif (Figure 3) [51,52].

**Scheme 1:**
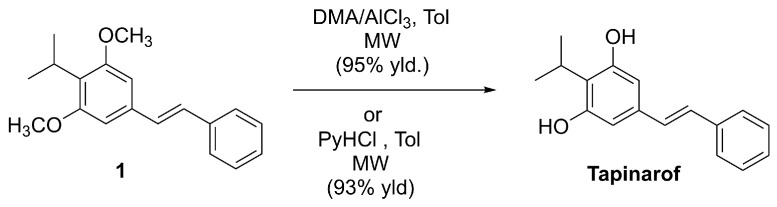
Synthesis of tapinarof from its dimethylated precursor **1** via microwave-assisted demethylation.

Although tapinarof was initially identified as a bacterial metabolite (reported in *Photorhabdus* spp. and related symbiotic bacteria) [52], its clinical and commercial development depends on scalable, well-controlled synthetic routes and robust impurity profiling. Accordingly, process patents describe industrially relevant preparations of tapinarof and the isolation of defined solid forms suitable for pharmaceutical use [53]. In parallel, several academic syntheses have been reported, including a practical late-stage demethylation of the (E)-3,5-dimethoxy precursor **1** (Scheme 1) under microwave irradiation using AlCl_3_/*N*,*N*-dimethylaniline or pyridinium chloride-based demethylating systems to furnish tapinarof (Scheme 1) [53].

Tapinarof engages NRF2 signaling indirectly, predominantly via AhR-driven pathways and AhR–NRF2 crosstalk in keratinocytes, thereby converging on NRF2/ARE cytoprotective programs (e.g., HO-1 and related stress-response enzymes) rather than through direct electrophilic modification of KEAP1 [54]. To date, direct covalent modification of KEAP1 cysteine residues by tapinarof has not been demonstrated. As a first-in-class topical AhR agonist, tapinarof induces transcriptional programs linked to epidermal differentiation and barrier reinforcement and modulates inflammatory signaling [2,4]. Consistent with an NRF2-focused framework, dual engagement of AhR- and NRF2-linked protective responses has been demonstrated in stress-relevant skin models [54,55], with NRF2 activation interpreted as secondary to AhR-driven transcriptional reprogramming. Clinically, tapinarof has demonstrated robust efficacy and favorable tolerability in pivotal Phase III trials in adults with plaque psoriasis and in both adult and pediatric patients with atopic dermatitis, supporting its approval for these indications. An additional Phase III clinical trial is currently ongoing to evaluate its efficacy and safety in the pediatric population with plaque psoriasis [56,57].

#### 4.1.2. Dimethyl Fumarate (DMF)

Dimethyl fumarate (DMF) is the dimethyl ester of fumaric acid (Figure 3) and represents one of the simplest fumaric acid esters used therapeutically.

It is synthesized by esterification of fumaric acid with methanol under acidic catalysis (commonly sulfuric acid), yielding the dimethyl ester in high purity and yield. This process is well established and scalable for pharmaceutical production. Continuous-flow synthesis approaches have recently been developed to improve process efficiency and sustainability, further supporting industrial manufacture [58,59].

DMF is one of the best-characterized electrophilic activators of NRF2. It reacts covalently with cysteine residues of the cytosolic NRF2 repressor KEAP1, particularly Cys151, as demonstrated in biochemical and mutational studies, causing conformational changes that inhibit NRF2 ubiquitination and degradation [60]. This mechanism explains the dual antioxidant and anti-inflammatory effects observed in keratinocytes, neurons, and immune cells. This canonical electrophilic mechanism positions DMF among the most mechanistically validated direct KEAP1-dependent NRF2 activators currently used in clinical practice.

DMF has a long clinical history in the systemic treatment of moderate-to-severe plaque psoriasis, particularly in Europe under the brand Fumaderm^®^. Its clinical efficacy is attributed to immune modulation, reducing Th1/Th17 cytokines, and NRF2-driven antioxidant defense, thereby normalizing keratinocyte proliferation and inflammatory pathways [61]. Preclinical evidence also suggests potential benefits in other oxidative stress-driven skin diseases such as atopic dermatitis and vitiligo, although robust clinical data are still lacking [62]. DMF is approved for systemic use in psoriasis and relapsing–remitting multiple sclerosis (Tecfidera^®^). Ongoing clinical and translational research is exploring its repurposing for other inflammatory and pigmentary skin diseases, and topical DMF formulations are being developed for localized skin application [62].

### 4.2. Approved Drugs and Supplements with Clinical Dermatology Use or Repurposing Potential

**Figure 4:**
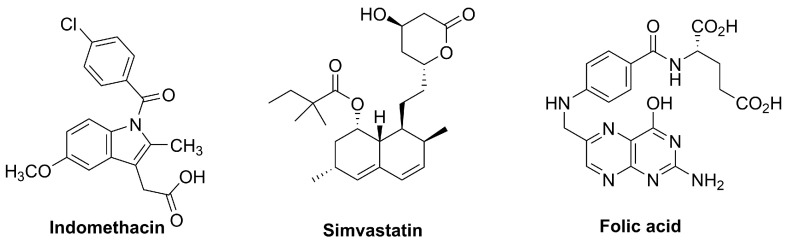
Chemical structures of approved drugs and supplements with clinical dermatology use or repurposing potential.

Compounds represented in Figure 4 are approved for other indications (or used as supplements) but have been evaluated clinically in dermatology and show NRF2-associated antioxidant or cytoprotective effects, supporting their consideration as repurposing/adjunct strategies.

#### 4.2.1. Indomethacin

Indomethacin is a well-established non-steroidal anti-inflammatory drug (NSAID) belonging to the indole acetic acid class. Its molecular structure consists of a substituted indole core linked to a chlorobenzoyl group (Figure 4), which confers potent cyclooxygenase (COX) inhibitory activity.

**Scheme 2:**
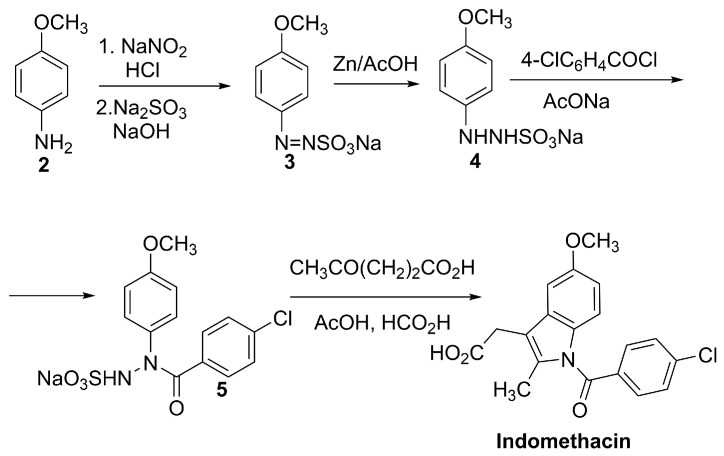
Indomethacin synthesis via Fischer indole synthesis.

The most common synthetic route involves N-acylation of the appropriate arylhydrazine precursor **4** with 4-chlorobenzoyl chloride to generate the amide **5**, prior to ring construction through a Fisher indole synthesis [63] (Scheme 2).

Beyond COX inhibition, indomethacin has been reported to modulate redox-sensitive signaling pathways, including activation of the KEAP1–NRF2 axis in experimental models. Mechanistically, indomethacin has been suggested to induce oxidative/electrophilic modifications of cysteine residues on KEAP1, which may inhibit KEAP1-mediated ubiquitination and proteasomal degradation of NRF2. This allows NRF2 to accumulate and translocate to the nucleus, where it binds antioxidant response elements (AREs) and drives the transcription of cytoprotective genes, including *Hmox1* (heme oxygenase-1) and *Nqo1* (NAD(P)H quinone dehydrogenase 1). This COX-independent mechanism was confirmed in murine myeloid cells using *Nrf2* knockout models, demonstrating that indomethacin’s anti-inflammatory effects partly rely on NRF2-dependent gene expression [64]. However, direct biochemical evidence of covalent KEAP1 modification by indomethacin remains limited, and NRF2 activation may represent a secondary consequence of broader redox modulation.

Topical formulations of indomethacin have been investigated in dermatology for their ability to attenuate UVB-induced erythema and inflammation. In controlled human and animal studies, topical indomethacin (2.5–5%) significantly reduced erythema following UVB exposure, likely through inhibition of prostaglandin synthesis and modulation of local inflammatory mediators [65]. These findings support its potential utility in photodamage, actinic keratosis, and other inflammatory dermatoses where prostaglandins and oxidative stress play a key role.

Indomethacin is widely approved for systemic use and is also available as topical formulations in several countries for localized inflammatory pain conditions. Although NRF2 pathway engagement has been reported for a subset of NSAIDs, the NRF2-modulating effects of indomethacin remain primarily supported by preclinical evidence [64]. To our knowledge, no registered clinical trials have specifically evaluated indomethacin as an NRF2-targeting intervention for oxidative stress–driven dermatological disorders.

#### 4.2.2. Simvastatin

**Figure 5:**
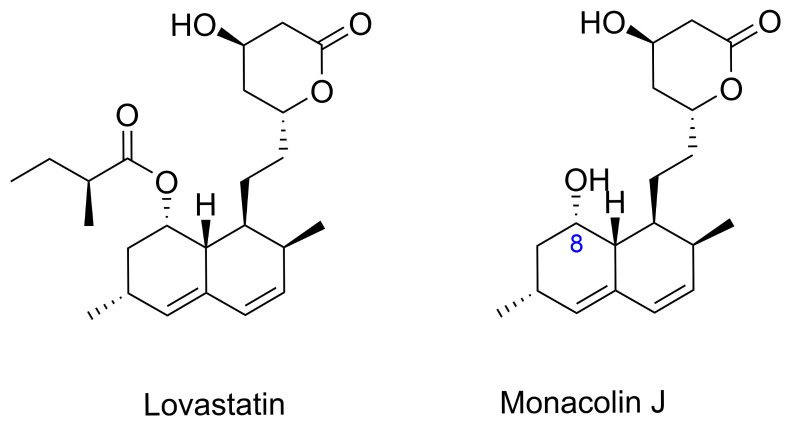
Structures of the simvastatin precursors, lovastatin and monacolin J.

Simvastatin is a semisynthetic derivative of lovastatin featuring a lactone ring essential for HMG-CoA reductase inhibition (Figure 4), which underlies its widespread use as a lipid-lowering agent in clinical practice [66]. At present, all commercially available simvastatin is derived either from this semisynthetic process or from biocatalytic transformation of fermentation-derived lovastatin or monacolin J (Figure 5).

Simvastatin is manufactured industrially using a semisynthetic pathway that begins with lovastatin, a metabolite produced by the fermentation of *Aspergillus terreus*. The conventional process involves hydrolysis of lovastatin to obtain monacolin J, followed by selective esterification at the C8 hydroxyl position (Figure 5). This approach streamlines production by eliminating the need for multiple protection and deprotection steps, and it remains the cornerstone of simvastatin manufacturing due to its efficiency and high yields [66,67]. Recent advances have focused on biocatalytic strategies, particularly those employing the acyltransferase LovD, which can be heterologously expressed in *Escherichia coli*. This enzyme facilitates the direct acylation of monacolin J with a 2,2-dimethylbutyryl donor in aqueous media, producing simvastatin in a single step. Ongoing efforts in directed evolution and protein engineering have enhanced the properties of LovD, improving its solubility, stability, and catalytic performance, thereby supporting sustainable and large-scale biocatalytic production of simvastatin [68].

Beyond its cholesterol-lowering effects, simvastatin has been reported to enhance the NRF2 pathway in melanocytes. The proposed mechanism includes inhibition of isoprenoid synthesis, leading to reduced prenylation of small GTPases and subsequent modulation of redox-sensitive signaling cascades that favor NRF2 nuclear translocation [69]. This results in increased expression of antioxidant enzymes, enhancing cellular resilience against oxidative stress [70]. In this context, NRF2 activation appears to be secondary to alterations in redox-sensitive signaling cascades rather than the result of direct electrophilic KEAP1 modification.

In dermatology, simvastatin has been explored as a repurposing candidate for vitiligo based on its NRF2-related antioxidant effects and anti-inflammatory activity. In vitro, it promotes NRF2 nuclear translocation in human melanocytes, increases antioxidant defenses, and may reduce oxidative stress–induced melanocyte loss [70]. However, a Phase II double-blind, placebo-controlled trial of oral simvastatin in vitiligo did not show a significant benefit versus placebo and reported adverse events (e.g., myalgia, elevated liver enzymes) [71]. Thus, despite encouraging mechanistic data, its clinical efficacy in vitiligo remains unproven.

#### 4.2.3. Folic Acid

Folic acid (pteroylmonoglutamic acid) is a synthetic, oxidized form of folate (vitamin B9), a water-soluble micronutrient required for one-carbon metabolism and therefore essential for DNA synthesis, repair, and methylation. Structurally, it comprises a pteridine ring linked to *p*-aminobenzoic acid (PABA) and conjugated to a glutamic acid residue (Figure 4).

**Scheme 3:**
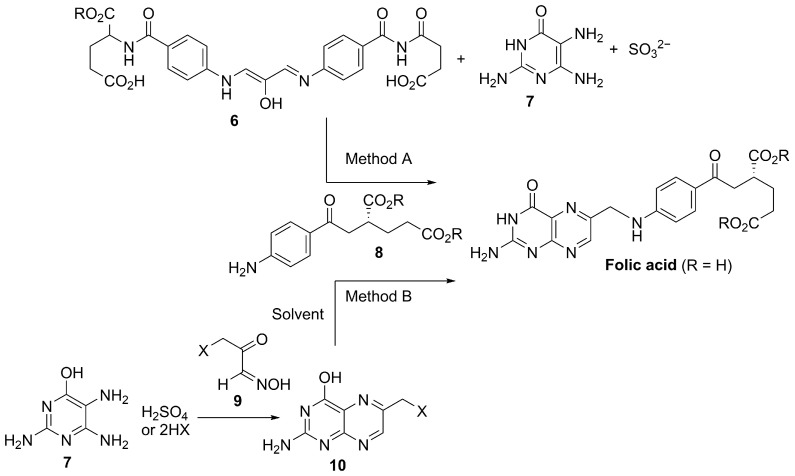
Representative patented syntheses of folic acid via a diimine intermediate **6** in the presence of sulfite (method A) or via halogenated pyruvaldehyde oxime intermediates **9** (method B) [72,73].

Industrial supply is based on multistep chemical synthesis, and patent literature describes optimized manufacturing routes. An improved process employs the diimine intermediate **6** that is converted to folic acid in the presence of sulfite over pH 3–8 (Scheme 3, method A), enabling substantially higher yields than earlier approaches [72]. Alternative patented methods have also been disclosed using halogenated pyruvaldehyde oxime intermediates **9** and aminopyrimidine salts under milder and potentially more environmentally friendly conditions (Scheme 3, method B) [73]. Although chemical synthesis remains the industrial standard, fermentation-based production has been explored as a greener option; for example, metabolic engineering of *Ashbya gossypii* through overexpression of folate-pathway genes has enabled folic acid production at mg/L–g/L titers [74].

Beyond its nutritional role, folic acid has attracted interest for antioxidant and cytoprotective effects in pigmentary disorders. In melanocyte models, folic acid has been reported to enhance resistance to oxidative stress, with mechanistic data consistent with NRF2 pathway engagement in cell-based experimental systems (including reduced *KEAP1* expression and increased NRF2 nuclear translocation) and downstream cytoprotective responses [75]. To our knowledge, the precise molecular mechanism linking folic acid to NRF2 activation remains incompletely defined, and direct biochemical evidence of KEAP1 cysteine modification has not been demonstrated. Clinically, folic acid is widely used as an oral supplement with a favorable safety profile and is often included in combination regimens for vitiligo. A clinical study reported improved repigmentation outcomes when oral vitamin B12 and folic acid were combined with sun exposure compared with either intervention alone; however, larger randomized controlled trials are still needed to establish efficacy and to define optimal dosing and treatment protocols [76].

### 4.3. Dermatology Clinical-Stage Candidates Targeting Oxidative Stress/NRF2-Related Pathways

**Figure 6:**
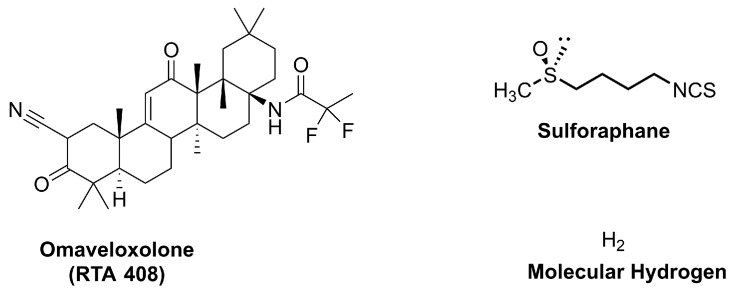
Structures of dermatology clinical-stage candidates targeting oxidative stress/NRF2-related pathways.

Compounds represented in Figure 6 have entered early clinical testing for dermatologic indications, providing proof-of-concept that pharmacologic engagement of oxidative-stress responses, including NRF2-linked programs, may translate into measurable skin benefit.

#### 4.3.1. Omaveloxolone (RTA 408)

**Figure 7:**
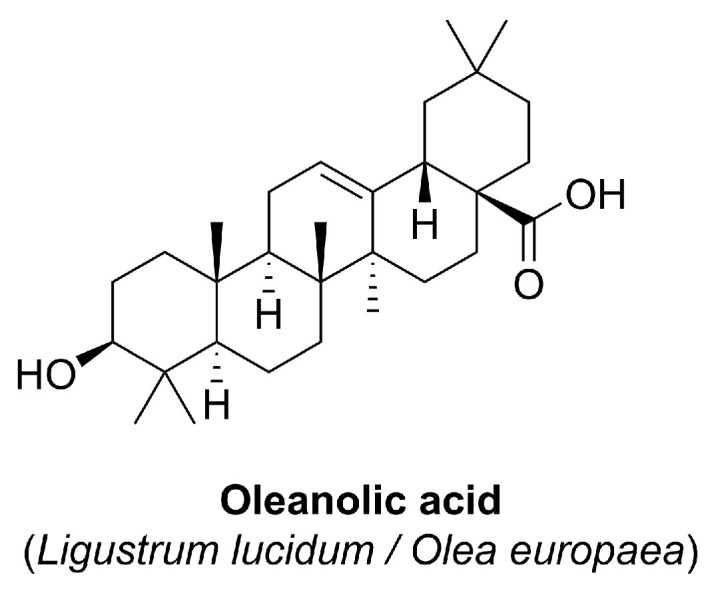
Structure of oleanolic acid, the natural precursor of omaveloxolone.

Omaveloxolone (Figure 6) is a semisynthetic derivative of oleanolic acid, a naturally occurring pentacyclic triterpenoid found in various medicinal plants such as *Ligustrum lucidum* and *Olea europaea* (Figure 7).

**Scheme 4:**
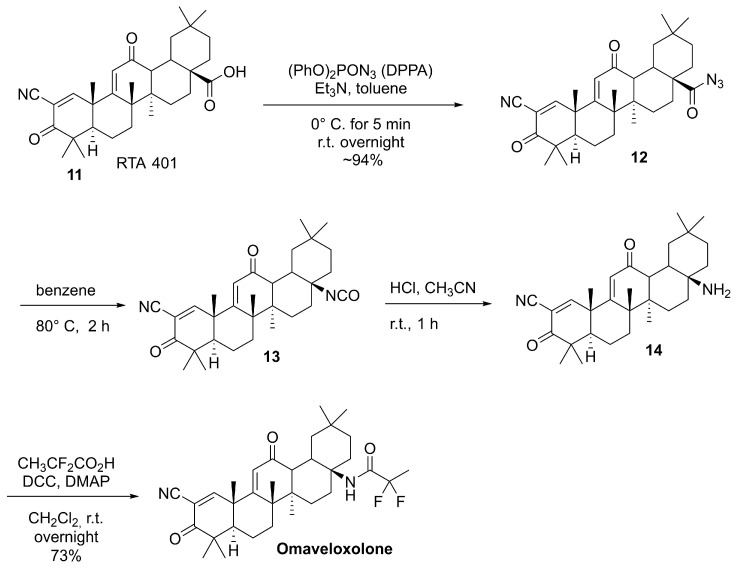
Semisynthesis of omaveloxolone from RTA 401.

The semisynthetic route to omaveloxolone is based on late-stage functionalization of the oleanane scaffold starting from RTA 401 (**11**, Scheme 4), a pre-functionalized oleanolic acid derivative, to introduce substituents that tune physicochemical and ADME properties. These modifications increase overall lipophilicity and metabolic stability while enhancing NRF2-activating potency, thereby improving suitability for topical and/or systemic administration relative to the parent natural product [77]. From a synthetic standpoint, the sequence proceeds through carboxylic acid activation of **11** using diphenylphosphoryl azide (DPPA) and triethylamine in toluene, generating the corresponding acyl azide **12**, which, upon heating, undergoes a Curtius rearrangement to the key isocyanate intermediate **13**. Subsequent transformation of **13** (via hydrolytic conversion) affords the primary amine **14**. Finally, **14** is derivatized by fluorinated acylation to deliver omaveloxolone [77].

The presence of activated alkenes as electrophilic Michael acceptor moieties in the structure facilitates covalent binding to cysteine residues on KEAP1, as demonstrated for related synthetic triterpenoids in biochemical and mutational studies, thereby promoting the NRF2 nuclear translocation and transcriptional activation of antioxidant and cytoprotective genes.

Preclinical studies demonstrated that topical application of omaveloxolone markedly attenuates radiation-induced dermatitis in murine models, reducing skin inflammation, ulceration, and epidermal thickening while enhancing expression of NRF2-dependent genes [78]. Early-phase clinical trials confirmed the safety and bioactivity of omaveloxolone in human skin, positioning it as a promising candidate for topical treatment of cutaneous radiation injury and other inflammatory skin conditions [79]. While its dermatological application is currently in Phase II with encouraging results, its systemic use has advanced further, receiving regulatory approval and continuing to be evaluated in pediatric development programs [80].

#### 4.3.2. Sulforaphane (SFN)

**Scheme 5:**
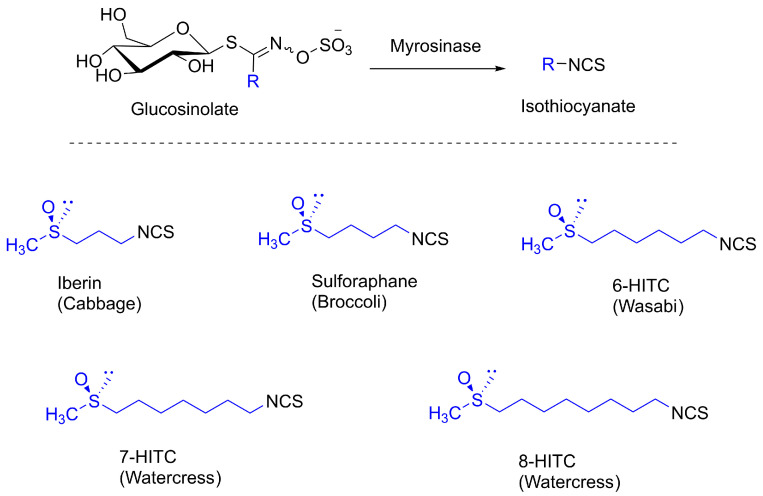
Natural isothiocyanates (sulforaphane and its homologs) derived from enzymatic hydrolysis of their glucosinolate precursors.

Sulforaphane (SFN), shown in Figure 6, [(*R*)-1-isothiocyanato-4-(methylsulfinyl)butane] is a structurally simple chiral sulfoxide first isolated in 1992 from broccoli extract [81]. Like other naturally occurring isothiocyanates, it is formed through the enzymatic hydrolysis of glucosinolates present in cruciferous vegetables, a process catalyzed by the enzyme myrosinase upon plant tissue disruption (Scheme 5) [82]. Among these vegetables, broccoli is especially rich in glucoraphanin, the stable aliphatic glucosinolate that serves as the direct biochemical precursor of SFN [81,83]. The molecule is structurally simple yet notable for containing a chiral sulfinyl group. As in other naturally occurring isothiocyanates, the sulfinyl sulfur in SFN exhibits an *R*-configuration, which results from a stereospecific oxidation catalyzed by flavin-containing monooxygenases [81].

Various synthetic and semisynthetic methods have been developed for SFN production, ranging from enzymatic hydrolysis to total chemical and asymmetric syntheses. In the context of semisynthesis, the biotransformation that naturally occurs in cruciferous vegetables, namely, the enzymatic hydrolysis of glucosinolates to isothiocyanates, can be reproduced in the laboratory to obtain high-yield production. A gram-scale, highly enantioselective method employs purified myrosinase from *Tuscan black kale* in a biphasic system to hydrolyze glucoraphanin, yielding enantiopure *R*-sulforaphane (>99% yield) (1.09 g of pure *R*-SFN from 150 g of defatted seed meal) [84].

**Scheme 6:**
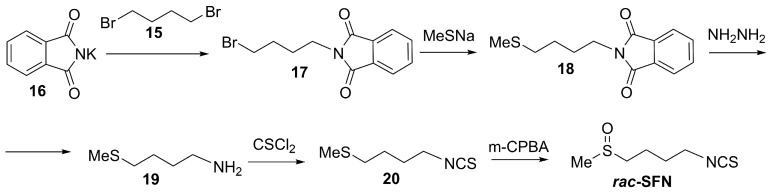
Synthesis of *rac*-SFN from 1,4-dibromobuthane **15** and potassium salt of phthalimide **16**.

Although SFN is defined by its chiral sulfoxide center, many syntheses have focused on producing it in racemic form. Racemic synthesis is simpler, more cost-effective, and enables the preparation of larger amounts of SFN compared to asymmetric routes that require chiral catalysts or auxiliaries. As an example of racemic synthesis of SFN, a practical multi-step approach starts from commercially available phthalimide salt **16** and 1,4-dibromobutane **15** (Scheme 6) [85,86]. The sequence begins with a Gabriel synthesis to form *N*-(4-bromobutyl) phthalimide **17**, followed by substitution with sodium methanethiolate to yield *N*-(4-(methylthio)butyl) phthalimide **18**. Subsequent hydrazinolysis releases 4-(methylthio)butylamine **19**, which undergoes thiophosgene-mediated transformation into the corresponding isothiocyanate **20**. Finally, oxidation of the thioether with m-CPBA furnishes racemic SFN in reliable yield.

**Scheme 7:**
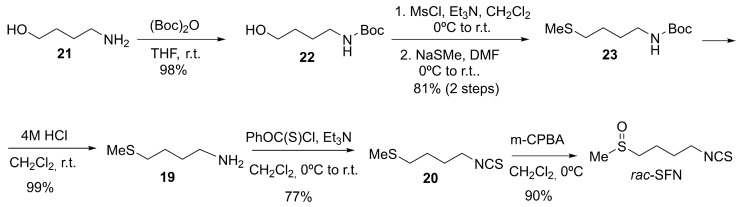
Synthesis of *rac*-SFN from 4-aminobutan-1-ol, **21**.

With the aim of improving previously described methodologies, Chen et al. developed an alternative route to racemic SFN starting from 4-aminobutan-1-ol, **21** [87]. This method avoids the use of toxic reagents such as thiophosgene, employs a mild one-pot conversion of the amine **19** to the isothiocyanate **20** using *O*-phenyl chlorothioformate, and achieves a higher overall yield (64%), making it suitable for multigram synthesis (Scheme 7).

**Scheme 8:**
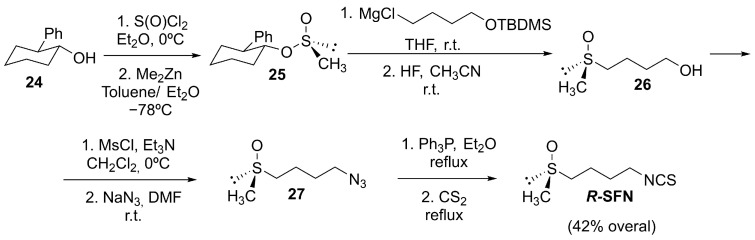
*R*-Sulforaphane synthesis via Whitesell’s chiral sulfinate ester method.

Regarding stereoselective synthesis, Whitesell and Wong reported a classic, versatile synthesis of both enantiomers of SFN via the chiral sulfinate ester derived from *trans*-2-phenylcyclohexanol [88]. Starting from the *trans*-(1*R*,2*S*) alcohol, **24**, through thionyl chloride and dimethyl zinc, they achieved the (*R*)-methane sulfinate **25** with a high stereochemical induction (92% *de*), which delivered *R*-sulforaphane in good yield (Scheme 8).

**Scheme 9:**
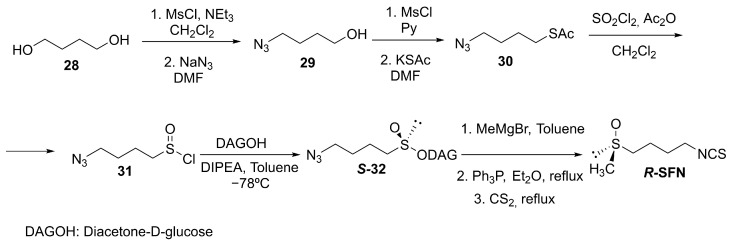
Application of the DAG methodology for the synthesis of *R*-SFN.

Building upon the diacetone-D-glucofuranose (DAG) chiral auxiliary strategy [89], our group synthesized the chiral sulfinate ester ***S*-32** as precursor and achieved stereoinversion via a Grignard-mediated pathway. Subsequent Staudinger/aza-Wittig transformation delivered *R*-SFN (Scheme 9) [90,91]. This methodology demonstrated excellent versatility, enabling the stereoselective preparation of structurally diverse SFN derivatives [92,93,94].

**Scheme 10:**
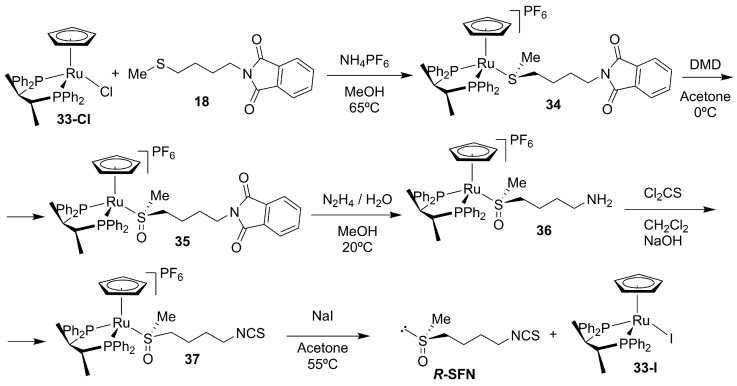
Synthesis of *R*-SFN using [CpRu((*R*,*R*)-CHIRAPHOS)]^+^ as chiral auxiliary.

Schenk and Dürr developed one of the first enantioselective syntheses of *R*-SFN using the ruthenium complex **33-Cl**, with the chiral bisphosphine ligand (*R*,*R*)-CHIRAPHOS as a stereodirecting auxiliary (Scheme 10) [95]. The sulfonium salt **34** was asymmetrically oxidized to the chiral sulfoxonium salt **35** with ~80% ee, which was subsequently converted into the isothiocyanate **37** through thiophosgene treatment, affording *R*-SFN after deprotection (Scheme 10). Although this method provided one of the first examples of catalytic stereocontrol in sulforaphane synthesis, its relatively modest enantioselectivity and multistep nature have limited its use compared to auxiliary-based or biocatalytic approaches.

**Figure 8:**
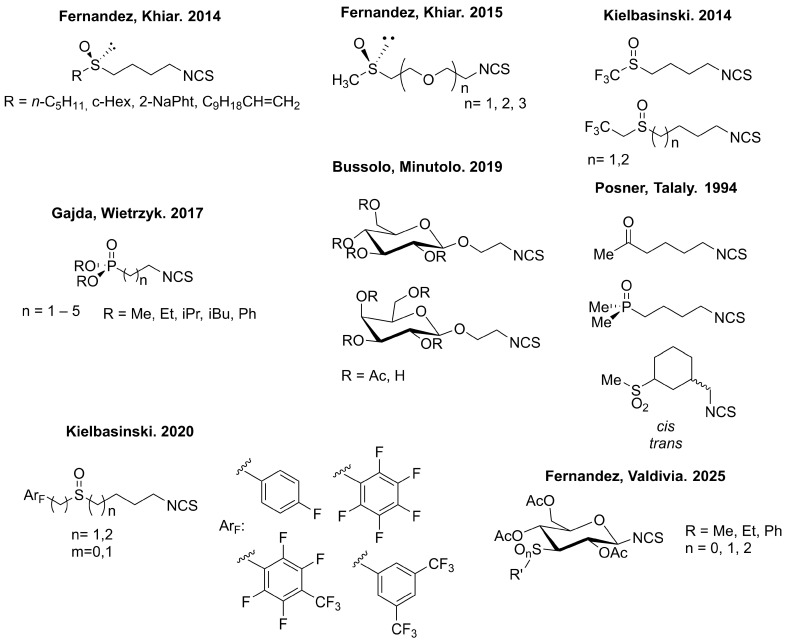
Some representative SFN analogs. The citations in the figures indicate the name of the researcher responsible for the study (the group’s principal investigator) [92,96,98,99,100,101,102,103].

Given the relative instability and liquid state of SFN, various research groups have focused on developing structurally related analogs with improved physicochemical properties [96,97,98,99,100,101,102,103]. These efforts aim to enhance stability, bioavailability, and potency, often favoring solid compounds more suitable for pharmaceutical development. The following figure illustrates the chemical structures of some of the most representative SFN analogs (Figure 8).

SFN activates the NRF2 pathway primarily through covalent modification of cysteine residues on KEAP1. This canonical electrophilic mechanism has positioned SFN as one of the most extensively characterized direct KEAP1-dependent NRF2 activators. SFN contains an electrophilic isothiocyanate group, which reacts with specific cysteines, particularly Cys151, Cys273, and Cys288, on KEAP1, as confirmed in biochemical and mutational studies using cysteine-targeted approaches.

SFN is recognized for its strong antioxidant and cytoprotective properties, which have attracted considerable interest in dermatological use, particularly in addressing photoaging and inflammatory skin diseases. Notably, SFN has demonstrated significant efficacy in shielding the skin from UV-induced damage and mitigating the effects of cutaneous aging [19,33].

The clinical development of SFN in dermatology is currently at an early stage, with promising results from both clinical and preclinical studies. A Phase I clinical trial (NCT03730649) at Johns Hopkins University is investigating the effects of topical SFN lotion on markers of skin aging and UV/visible light exposure in healthy adults; although the sample size is small, this represents an important step toward clinical application [104]. Nevertheless, dermatology-specific clinical evidence remains limited, and most mechanistic data derive from preclinical or systemic studies. In addition, a completed Phase II study (NCT00982319) assessed oral broccoli sprout extract, rich in SFN, for its systemic antioxidant effects, providing further rationale for its use in skin health, even though this trial was not dermatology-specific [105]. Preclinical research supports these findings, as numerous in vitro and animal studies have shown that SFN can protect against UV-induced skin damage, reduce inflammation and oxidative stress, and ameliorate features of atopic dermatitis by activating the NRF2/HO-1 axis and reducing immune cell infiltration and IgE levels [106]. These data together highlight the growing interest and therapeutic potential of SFN for dermatological applications. It remains one of the most advanced natural NRF2 activators being explored for dermatological indications.

#### 4.3.3. Molecular Hydrogen (H_2_)

It is worth noting that molecular hydrogen (H_2_, Figure 6), although not synthesized in the conventional chemical sense for therapeutic purposes, can be administered through various delivery systems, including hydrogen-rich water, hydrogen gas inhalation, and topical hydrogen-enriched solutions [107]. These methods have been developed to introduce molecular hydrogen into biological systems, where it can exert selective antioxidant and cytoprotective effects.

Molecular hydrogen (H_2_) has been reported to modulate NRF2 indirectly by reducing oxidative stress, thereby favoring NRF2 stabilization and nuclear accumulation. In dermatology-relevant models, hydrogen-rich media protect keratinocytes and melanocytes under oxidative challenge by lowering intracellular ROS and limiting apoptosis, consistent with reinforcement of endogenous antioxidant defenses. Importantly, molecular hydrogen does not function as an electrophilic KEAP1 modifier, and direct cysteine modification has not been demonstrated. NRF2 activation in this context is therefore interpreted as secondary to redox modulation rather than canonical KEAP1-dependent sensing. In human melanocytes, H_2_ has been reported to enhance NRF2 signaling in cellular models; however, covalent KEAP1 modification has not been demonstrated [108,109].

Molecular hydrogen is currently being explored in both preclinical and early-phase clinical studies for a range of oxidative and inflammatory diseases [110]. In dermatology, its use remains experimental, with most data coming from in vitro and animal models. However, its favorable safety profile and antioxidant properties make it a promising candidate for adjunctive therapy in conditions like vitiligo. Current evidence remains largely preclinical or exploratory, and controlled dermatology-specific clinical validation is still limited.

### 4.4. Preclinical Dermatology Candidates with Defined Structures and NRF2-Linked Mechanisms

**Figure 9:**
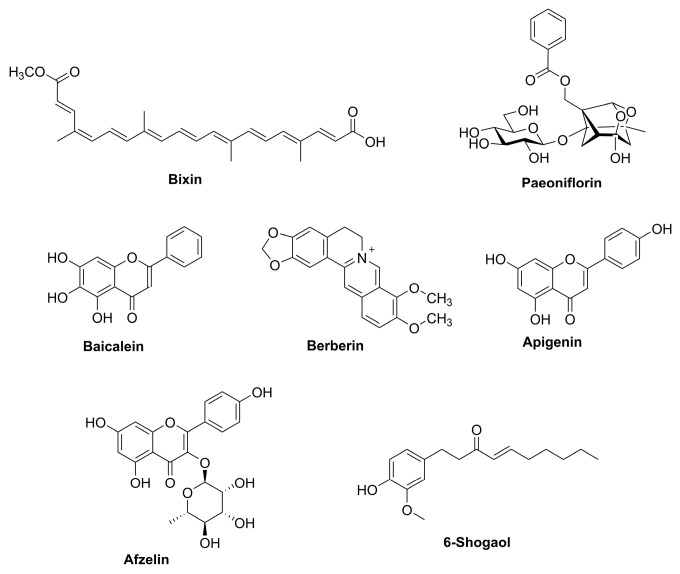
Structures of preclinical dermatology candidates with NRF2-linked mechanisms.

In this section, we describe several chemically defined compounds (Figure 9) that have shown compelling preclinical efficacy in skin-relevant models and activate NRF2 either directly (e.g., via KEAP1 modulation) or indirectly through upstream signaling pathways; together, they constitute a promising pipeline for future topical and/or systemic development.

For most of the compounds discussed in this section, NRF2 pathway engagement has been demonstrated primarily in cell-based or animal models. In several cases, direct biochemical validation of KEAP1 cysteine modification is lacking; therefore, NRF2 activation should be interpreted as indirect or context-dependent.

#### 4.4.1. Bixin

Bixin is a structurally defined apocarotenoid (C25) (Figure 9) and the major lipophilic pigment of annatto (*Bixa orellana*), widely used as a natural colorant. In practice, bixin is commonly obtained by extraction and purification from annatto preparations rather than by de novo total synthesis [111].

Beyond its pigmentary applications, bixin has emerged as a compound with robust NRF2 pathway engagement and strong in vivo dermatological relevance. In murine models of solar UV exposure, systemic administration of bixin attenuated acute photodamage, including oxidative DNA damage and inflammatory responses, and genetic validation showed that protection is NRF2-dependent [112]. Mechanistically, bixin has been reported to activate NRF2 via the canonical KEAP1 sensor mechanism involving Cys151, consistent with an electrophile-driven mode of NRF2 stabilization and ARE gene induction [22,113]. Although NRF2 dependence has been genetically validated in vivo, direct biochemical demonstration of covalent KEAP1 cysteine adduct formation by bixin remains limited in skin-specific systems. Complementing systemic studies, topical bixin has also been shown to confer NRF2-dependent protection against UV-induced photodamage and to mitigate stress-induced hair graying in a PUVA-related model, supporting its potential as a topical cytoprotective agent [5,23,114]. Collectively, bixin exemplifies an NRF2-activating small molecule with dermatological in vivo validation and provides a rationale for photoprotective strategies that enhance endogenous antioxidant defenses beyond conventional UV filtering [112,114].

#### 4.4.2. Paeoniflorin

Paeoniflorin (PF) is a monoterpene glycoside primarily isolated from the roots of *Paeonia* species, especially *Paeonia lactiflora* and *Paeonia suffruticosa*. It is biosynthesized in plants via the terpenoid pathway and consists of a pinane skeleton glycosylated at the C-1 position with a β-D-glucopyranosyl moiety (Figure 9) [115].

**Scheme 11:**
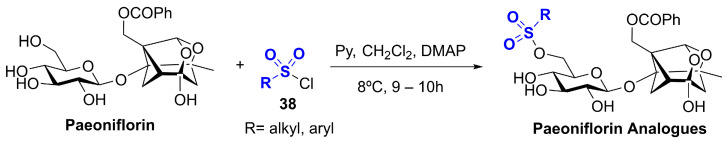
Synthetic route of paeoniflorin analogs.

The traditional method for obtaining PF involves solvent-based extraction from dried *Paeonia lactiflora* roots, followed by chromatographic quantification, although concerns about solvent toxicity and yield variability persist. To address these limitations, green extraction strategies such as the use of deep eutectic solvents (DESs) have been proposed, achieving improved selectivity and sustainability [116]. Comparative transcriptomic analyses across distinct *P. lactiflora* genotypes have identified candidate genes associated with terpenoid backbone and monoterpenoid biosynthesis, revealing genotype- and organ-dependent differences in upstream precursor pathways and monoterpene synthase expression, and providing a resource for future metabolic engineering efforts [113]. MicroRNA (miRNA) regulatory networks have been implicated in the tissue-specific regulation of PF accumulation and monoterpenoid-related pathways in *P. lactiflora*, highlighting an additional post-transcriptional layer of control within secondary metabolism [117]. In parallel, microbial biotransformation has emerged as a complementary approach: a paeoniflorin-converting enzyme from *Cunninghamella blakesleeana* has been identified and functionally characterized, supporting the feasibility of enzyme-based conversion routes that could be leveraged for scalable biocatalytic processing [118]. Recent advances have enabled the semisynthetic modification of PF, with several novel derivatives synthesized to enhance its antioxidant and pharmacological potential through targeted structural modifications, including reacting PF with various sulfonyl chlorides **38** (Scheme 11) [119].

PF activates the NRF2 signaling pathway primarily through modulation of redox-sensitive upstream targets. Experimental evidence suggests that PF may disrupt the KEAP1–NRF2 complex by inducing oxidative or electrophilic modifications of KEAP1, thereby inhibiting NRF2 degradation. Additionally, PF may engage kinase-mediated mechanisms, such as PI3K/Akt or MAPK pathways, that influence the phosphorylation status of NRF2, facilitating its stabilization and activation. In a murine model of ulcerative colitis, PF treatment markedly increased NRF2 and HO-1 expression while reducing oxidative stress markers, supporting NRF2 pathway engagement consistent with upstream redox- and kinase-mediated mechanisms [120]. Direct biochemical evidence of covalent *KEAP1* cysteine modification by paeoniflorin has not been demonstrated, and NRF2 activation is therefore best interpreted as indirect and context-dependent.

Although direct clinical development of PF for dermatologic indications is still limited, mechanistic studies support its relevance in redox- and inflammation-driven epithelial injury. PF attenuated UVA-associated oxidative stress through NRF2 signaling in skin-relevant cellular settings [121] and inhibited NLRP3 inflammasome activation via NRF2/HO-1 in a Sjögren’s syndrome model, pointing to a broader anti-inflammatory stress-adaptive mechanism [122]. In addition, PF reduced NF-κB/NLRP3–linked inflammation and improved outcomes in diabetic foot ulcer models, including keratinocyte-relevant in vitro systems [123]. Clinically, evidence is available mainly for Total Glucosides of Peony preparations (with paeoniflorin as a major constituent), which showed efficacy and safety in a multicenter, randomized, double-blind, placebo-controlled trial in primary Sjögren’s syndrome [124] and reduced hepatotoxicity when co-administered with methotrexate and leflunomide in rheumatoid arthritis studies [125,126].

#### 4.4.3. Baicalein

**Figure 10:**
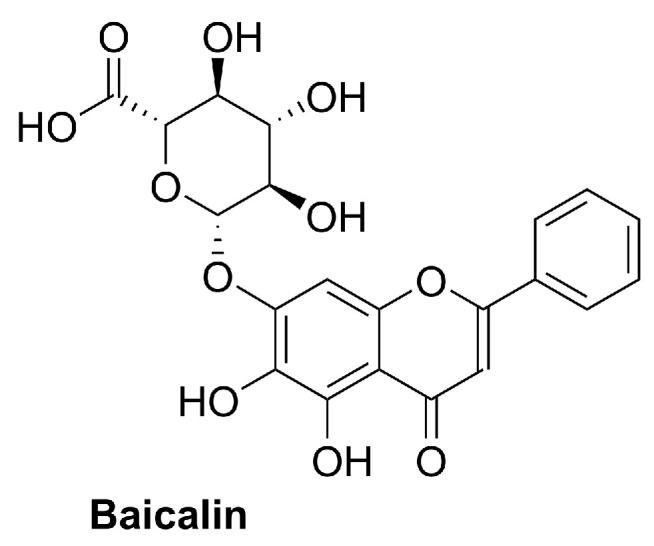
Structure of baicalin.

Baicalein (5,6,7-trihydroxy-2-phenyl-4H-1-benzopyran-4-one; Figure 9) is a naturally occurring flavone with a planar 2-phenylchromen-4-one scaffold bearing hydroxyl groups at C-5, C-6, and C-7, a substitution pattern associated with strong polyphenolic antioxidant activity. It is mainly obtained by extraction from *Scutellaria* species (*Lamiaceae*), particularly *Scutellaria baicalensis* and *Scutellaria lateriflora*, where it occurs as a major bioactive constituent together with its glycoside baicalin (Figure 10) [127,128]. Baicalein has also been reported in other botanical sources, including *Oroxylum indicum* [129], and has been isolated from *Thymus vulgaris* (*thyme*) [130].

**Scheme 12:**
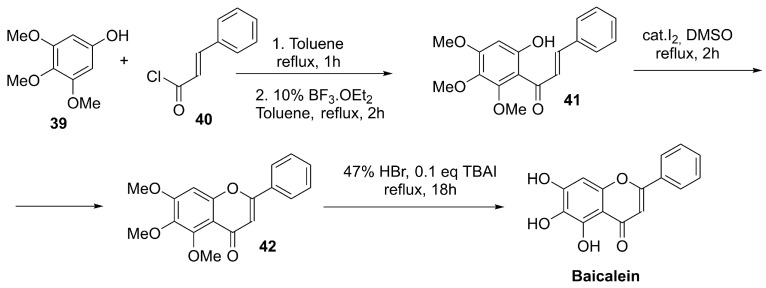
Scalable preparation of baicalein through a Fries rearrangement step followed by TBAI-promoted demethylation (HBr, reflux).

In plants, baicalein is produced through the flavonoid pathway. In the 4-deoxyflavone branch characteristic of *Scutellaria roots*, chrysin (5,7-dihydroxyflavone) is generated by flavone synthase and can subsequently undergo enzymatic 6-hydroxylation (flavone 6-hydroxylase) to yield baicalein. Although extraction from *Scutellaria* species remains the main source for experimental use, synthetic approaches have been developed to secure larger quantities. For multigram supply, Kim et al. fine-tuned a reported route by lowering the loading of a strong Lewis acid in the key Fries rearrangement and using tetrabutylammonium iodide (TBAI) as an additive to accelerate demethylation of precursor **42** in refluxing HBr, enabling preparation of >10 g of baicalein (Scheme 12) [131].

**Scheme 13:**
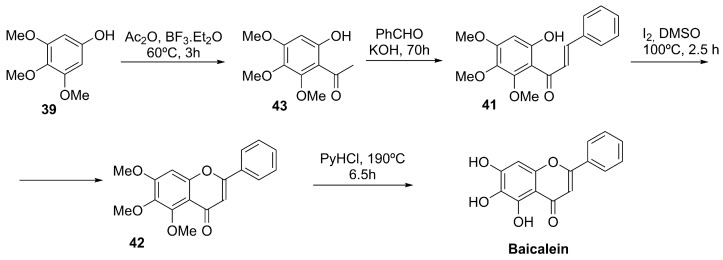
Synthesis of baicalein via benzaldehyde condensation and I_2_/DMSO cyclization.

Using also trimethoxyphenol **39** as a starting material, Chen et al. described a total synthesis involving the condensation of the ketone derivative **43** with benzaldehyde, followed by intramolecular cyclization (I_2_/DMSO) of the α,β-unsaturated aromatic ketone **41** and high-temperature demethylation of chromenone **42**, affording baicalein in 59% overall yield (Scheme 13) [132].

In vitiligo-relevant human melanocytes exposed to H_2_O_2_, baicalein has been reported to activate NRF2, promoting nuclear translocation and antioxidant defenses, which lower ROS and apoptosis; these effects are abolished by NRF2 silencing and appear independent of ERK or PI3K/Akt signaling [133]. Although NRF2 dependence is supported by silencing experiments, direct biochemical evidence of covalent KEAP1 cysteine modification in skin-relevant systems remains limited. More recent evidence suggests baicalein may also weaken KEAP1–NRF2 binding via direct interaction with KEAP1, consistent with Michael-acceptor reactivity [134].

Topical baicalein reduced disease severity and inflammatory mediators in an NC/Nga mouse model of atopic dermatitis induced by *Dermatophagoides pteronyssinus* [135], and oral administration limited acute UVB-induced skin thickening while suppressing MMP-9/VEGF and COX-2/NF-κB signaling in hairless mice, consistent with photoprotective activity [124]. Overall, dermatology-focused evidence remains preclinical and derives from in vitro studies and animal models (e.g., vitiligo-relevant oxidative stress, atopic dermatitis, and UV injury) [133,134,135,136]. In humans, oral baicalein tablets have shown acceptable safety and predictable pharmacokinetics in a Phase I multiple-ascending-dose study in healthy volunteers [137], and a Phase II randomized, placebo-controlled trial has been registered (NCT03830684) for a non-dermatologic indication, with no results posted to date [138].

#### 4.4.4. Berberine

Berberine is a natural isoquinoline alkaloid characterized by a planar, quaternary ammonium structure (Figure 9), and it is primarily extracted from the roots, rhizomes, and bark of plants such as *Berberis vulgaris* and other species of the *Berberidaceae* family [139].

**Scheme 14:**
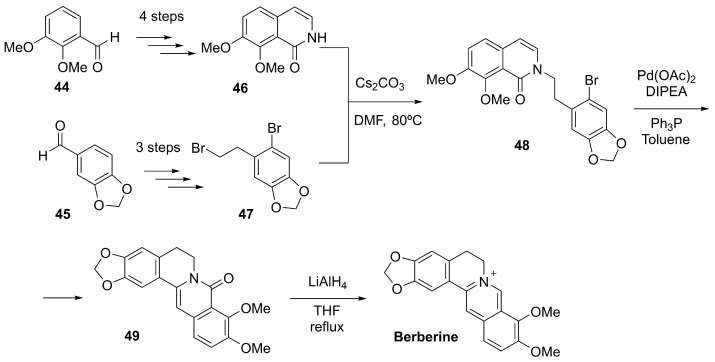
Total synthesis of berberine via Heck coupling.

While berberine has traditionally been obtained from medicinal plants, several efficient laboratory synthesis methods have also been established. Tajiri et al. achieved the total chemical synthesis of berberine (and related analogs) using an intramolecular Heck coupling of **48** as the central step to construct the isoquinolinium core. After final reduction in the amide **49**, this approach enabled the generation of fully synthetic berberine (Scheme 14) [140].

**Scheme 15:**
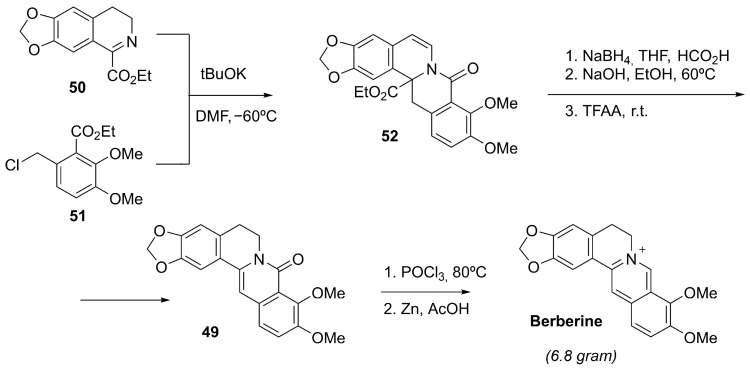
Gram scale total synthesis of berberine.

Subsequently, Yan et al. patented an alternative scalable, convergent synthesis of berberine hydrochloride employing a trifluoroacetic anhydride–promoted decarbonylative elimination of the intermediate **52**, facilitating gram-scale production without requiring complex purification procedures (Scheme 15) [141].

Berberine has been reported to enhance NRF2 pathway activity, at least in part, by alleviating intracellular oxidative stress; accordingly, NRF2 silencing in human melanocytes markedly reduces berberine-mediated protection against H_2_O_2_-induced damage [142]. In vitro, berberine has shown relevance for pigmentary disorders such as vitiligo by engaging both the NRF2/ARE and melanogenic programs. In PIG1 melanocytes exposed to H_2_O_2_, berberine increased NRF2 nuclear translocation, boosted antioxidant defenses (e.g., HO-1 and SOD), and lowered ROS levels and apoptosis, while also enhancing melanogenesis through upregulation of *MITF* and its target genes in an NRF2-dependent manner [142]. NRF2 activation in this context appears to result from modulation of upstream redox-sensitive signaling rather than direct electrophilic KEAP1 modification. Although clinical evidence is still lacking, topical delivery systems (e.g., berberine-loaded hyalurosomes) have shown activity in preclinical vitiligo models, improving biochemical readouts and pigmentation-related gene expression [143].

#### 4.4.5. Apigenin

Apigenin (4′,5,7-trihydroxyflavone) (Figure 9) is a naturally occurring flavonoid widely found in plants such as parsley, chamomile, and celery, as well as various other fruits and vegetables. In plants, it is biosynthesized via the phenylpropanoid pathway, starting from phenylalanine, which is transformed through a series of enzymatic steps involving phenylalanine ammonia-lyase (PAL), chalcone synthase (CHS), and flavone synthase (FNS) to yield the core flavone structure [144].

In addition to its natural production, apigenin can also be synthesized through chemical strategies, such as the cyclization of chalcone intermediates or targeted hydroxylation of flavone precursors, approaches that are useful in pharmaceutical development [145]. Nevertheless, the most widely used method remains plant-based extraction, employing both classical solvent techniques (e.g., ethanol, methanol) and more sustainable technologies such as ultrasound-assisted extraction, supercritical fluid extraction, and microwave-assisted extraction [146,147].

Apigenin has been reported to enhance NRF2 signaling activity in experimental models primarily by interfering with its cytoplasmic repressor KEAP1, leading to NRF2 stabilization and release [148]. In addition, apigenin has been shown to activate upstream kinases such as ERK and PI3K/Akt, which in turn phosphorylate NRF2, further promoting its dissociation from KEAP1 [149]. It has also been demonstrated that apigenin increases intracellular reactive oxygen species (ROS) in a controlled manner, serving as a redox signal to initiate KEAP1-NRF2 uncoupling without causing cytotoxicity [150,151]. While NRF2 nuclear translocation and downstream antioxidant gene induction have been observed, a canonical KEAP1 cysteine-sensing mechanism has not been formally established.

These multifaceted interactions enable apigenin to act as a robust NRF2 activator across different tissues, particularly in settings of oxidative stress [152]. In dermatology, apigenin is therefore of interest for conditions in which redox imbalance and melanocyte dysfunction contribute to disease pathogenesis. In vitiligo-relevant models, apigenin has been reported to engage the NRF2 pathway and reinforce antioxidant defenses, thereby limiting ROS-driven melanocyte apoptosis. In addition, it may help preserve pigment cell homeostasis by modulating redox-sensitive signaling within the skin microenvironment, including pathways implicated in the redox-sensitive regulation of melanocyte homeostasis in this niche [153].

At present, apigenin remains at a preclinical stage for dermatological indications. While multiple in vitro and animal studies support its bioactivity and tolerability, to date no dermatology-specific clinical trials have been registered. Nevertheless, apigenin is already available in cosmetic products and dietary supplements, reflecting its favorable safety profile and broad biological activity [154].

#### 4.4.6. Afzelin

Afzelin, 4′,5,7-trihydroxy-3-(α-D-rhamnopyranosyloxy) flavone, is a naturally occurring flavonol glycoside, specifically the 3-*O*-rhamnoside of kaempferol (Figure 9). It is commonly found in various plant species, particularly those rich in kaempferol derivatives, such as *Prunus persica*, *Fagopyrum esculentum*, and *Elaeagnus umbellata*. These natural matrices constitute the primary source of afzelin for pharmacological studies, with the compound occurring as a minor constituent in polar flavonoid-enriched fractions [155,156,157].

Despite its natural abundance, the increasing demand for standardized and reproducible sources has prompted the exploration of alternative production methods. Biotechnological approaches, particularly those involving glycosyltransferase-mediated glycosylation, offer a viable route for the biosynthesis of afzelin and structurally related flavonol glycosides. Plant UDP-glycosyltransferases (UGTs), which catalyze the regioselective transfer of sugars to specific hydroxyl groups of flavonoids, have been studied extensively in this context. Kaempferol-specific UGTs capable of catalyzing 3-*O*-rhamnosylation represent promising tools for the generation of afzelin analogs under controlled conditions [158].

Although afzelin has not yet been synthesized via total chemical synthesis in an industrial context, chemoenzymatic strategies and recombinant UGT platforms are under active investigation. These systems aim to enable scalable, environmentally benign production routes, especially for applications requiring consistent bioactive profiles in pharmaceutical and cosmeceutical formulations [159].

Afzelin has been shown to indirectly activate the NRF2 pathway through inhibition of glycogen synthase kinase 3 beta (GSK-3β), a negative regulator of NRF2. By suppressing GSK-3β, afzelin prevents the phosphorylation and subsequent nuclear export of NRF2, thereby increasing its nuclear accumulation and activation of target antioxidant genes. This mechanism is consistent with KEAP1-independent stabilization of NRF2 rather than canonical cysteine-sensing modification of KEAP1. In pigment cell models related to vitiligo, afzelin has demonstrated protective effects against oxidative stress. Its capacity to enhance NRF2 signaling contributes to the maintenance of redox balance and preservation of melanocyte function [160].

Currently, the application of afzelin in dermatology remains at the preclinical stage. Its effects have been demonstrated in vitro, but there are no clinical trials or commercially available dermatological products containing afzelin to date [160]. Further studies are needed to validate its pharmacokinetics, formulation, and efficacy in vivo.

#### 4.4.7. 6-Shogaol

**Scheme 16:**
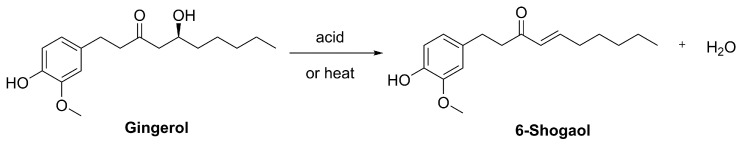
Semisynthetic route of 6-shogaol from 6-gingerol.

6-Shogaol, (4*E*)-1-(4-Hydroxy-3-methoxyphenyl)dec-4-en-3-one (Figure 9), is a bioactive compound derived from gingerol, the principal pungent component of fresh ginger (Scheme 16).

6-Shogaol can be obtained either through semisynthesis or through total chemical synthesis starting from simpler aromatic precursors. Both pathways are relevant for pharmacological development and structural modification. In semisynthesis, 6-shogaol is typically obtained through acid- or heat-induced dehydration of 6-gingerol, a reaction that naturally occurs during processing and involves the removal of a water molecule from the β-hydroxy ketone moiety to form the α,β-unsaturated ketone (Scheme 16) [161,162]. A more sophisticated and environmentally friendly approach has been developed, employing the acidic ionic liquid 1-butyl-3-methylimidazolium hydrosulfate ([Bmim]HSO_4_) under ultrasound irradiation to achieve the dehydration more efficiently and sustainably [163].

**Scheme 17:**
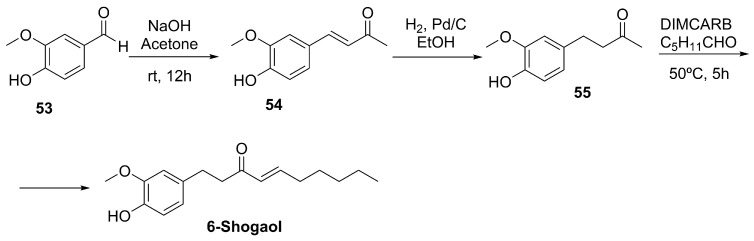
Total chemical synthesis of 6-shogaol.

Alternatively, 6-shogaol can be synthesized de novo starting from vanillin **53**. The typical pathway involves a Claisen–Schmidt condensation between vanillin and acetone to produce dehydrozingerone **54**, followed by reduction, aldol condensation with hexanal, and dehydration steps to yield 6-shogaol (Scheme 17) [164].

**Scheme 18:**
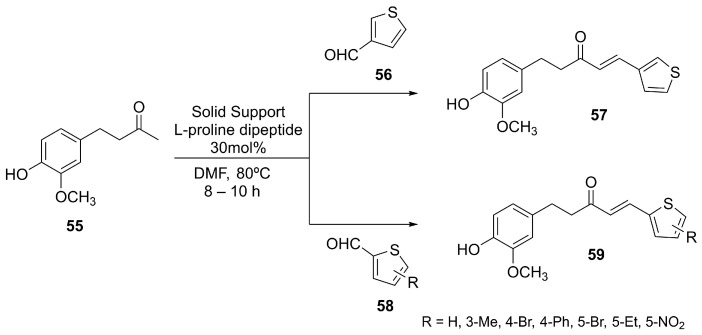
Synthesis of shogaol thiophene analogs, **57** and **59**.

To improve stability and pharmacological performance, synthetic analogs of 6-shogaol have been developed via thiophene derivatization (Scheme 18), considering that the vanillyl and the α,β-unsaturated carbonyl groups present in 6-shogaol are considered essential pharmacophores responsible for its biological activity [165]. The prepared shogaol thiophene compounds (STCs) (**57** and **59**, Scheme 18) showed anti-inflammatory effects via NRF2-dependent and -independent mechanisms. The STCs exhibited lower reactivity with thiols than 6-shogaol, suggesting they may cause fewer side effects and display greater metabolic stability.

Mechanistically, 6-shogaol, as an electrophilic molecule, is proposed to modify cysteine residues on KEAP1 via covalent bond formation, thereby releasing NRF2 and promoting the transcription of antioxidant response genes. Although its electrophilic structure is consistent with KEAP1 cysteine targeting, direct biochemical demonstration of covalent KEAP1 adduct formation in dermatologic models remains limited.

6-Shogaol is currently in preclinical development, with no registered clinical trials for dermatological use to date. In vitro studies using human epidermal melanocytes (HEMn-MPs) have demonstrated that 6-Shogaol activates the NRF2/ARE pathway, upregulates antioxidant enzymes (such as HO-1 and NQO1), and protects against oxidative stress–induced cell damage, including challenges from H_2_O_2_ and rhododendrol. These findings underscore its potential for preventing melanocyte loss in vitiligo [166]. While these results are promising, future work is necessary, including in vivo efficacy studies and eventual clinical trials, to fully establish its role in the treatment of pigmentary disorders.

To facilitate translational interpretation and comparative evaluation of NRF2-modulating compounds discussed above, a summary of their chemical class, mechanism of NRF2 engagement, dermatologic indications, level of evidence, and regulatory status is provided in Table 2.

## 5. Conclusions

Pharmacological modulation of the KEAP1–NRF2–ARE pathway represents a promising, mechanism-based strategy for dermatological conditions in which oxidative stress and chronic inflammation contribute to disease initiation or persistence. The evidence discussed in this review indicates that diverse natural, semisynthetic, and synthetic compounds can reinforce endogenous cytoprotective programs, support epidermal barrier resilience, and improve the stress tolerance of key cutaneous cell types, including keratinocytes and melanocytes. Importantly, the translational landscape is heterogeneous: while some NRF2 modulators have reached advanced clinical development and established therapeutic use, many others remain supported primarily by preclinical data despite encouraging in vitro and in vivo findings. Moving forward, rigorous, well-controlled clinical studies will be essential to define clinical efficacy, long-term safety, and the optimal magnitude, duration, and tissue localization of NRF2 engagement—particularly for topical delivery, where maximizing local benefit while minimizing systemic exposure is more readily achievable. Overall, targeting NRF2 emerges as a versatile approach with potential value across a spectrum of oxidative stress–related skin disorders, including photoaging, pigmentary alterations, inflammatory dermatoses, and cutaneous injury states, provided that pathway activation is appropriately tuned to disease context and treatment goals.

## 6. Future Directions

Despite substantial advances in understanding the KEAP1–NRF2–ARE axis in skin biology, important translational gaps remain before NRF2-targeted approaches can be broadly and safely implemented in dermatology. A major priority is to better resolve cell-type-specific NRF2 programs across keratinocytes, melanocytes, fibroblasts, and cutaneous immune cells, as these cell types likely differ in redox thresholds and downstream transcriptional outputs under inflammatory, pigmentary, fibrotic, and neoplastic conditions. In this context, combining disease-relevant models with high-resolution molecular profiling may help clarify when NRF2 activation is truly corrective versus potentially maladaptive.

Defining disease-specific therapeutic windows remains equally important. As emphasized in this review, insufficient NRF2 signaling can contribute to oxidative vulnerability and persistent inflammation, whereas prolonged or excessive activation may favor hyperproliferation, altered differentiation, or tumor-supportive stress adaptation in certain settings. Future pharmacological development should therefore prioritize control over the magnitude, duration, and tissue localization of pathway engagement. Topical delivery and formulation strategies that promote localized, time-limited NRF2 activation are particularly attractive for maximizing benefit while limiting systemic exposure.

Progress in clinical translation will also depend on the development of robust biomarkers of NRF2 pathway engagement. Practical pharmacodynamic readouts, such as ARE-driven gene expression signatures (e.g., HO-1, NQO1 and related targets) in skin samples, could support patient stratification, dose optimization, and early detection of excessive pathway activation. Incorporating such biomarkers into early clinical studies would strengthen mechanistic attribution and improve interpretability of clinical outcomes.

Long-term safety evaluation remains essential, especially for chronic dermatoses that may require prolonged treatment and for individuals with extensive UV exposure or premalignant lesions. Given the well-recognized context dependence of NRF2 in carcinogenesis, future studies should explicitly consider exposure duration, cumulative dose, and patient risk factors when evaluating NRF2-activating therapies.

Finally, rational combination strategies may expand therapeutic utility while improving tolerability. NRF2 modulation could be explored alongside barrier-repair agents, targeted anti-inflammatory therapies, phototherapy, or other redox-modulating interventions, with the goal of achieving efficacy at lower individual drug exposures. Overall, future research should move beyond generalized antioxidant paradigms toward disease-stratified, biomarker-guided NRF2 modulation that aligns pathway engagement with the specific biology and treatment goals of each dermatologic indication.

## Figures and Tables

**Table 1:** Acute vs. Chronic NRF2 Activation in Skin.

Feature	Acute Activation	Chronic Hyperactivation
Duration	Transient	Sustained
Functional outcome	Cytoprotection	Hyperproliferation/tumor support
Keratinocytes	Barrier reinforcement	Stress keratin induction
Melanocytes	Cell survival	Potential melanoma support
Fibroblasts	Wound repair support	Fibrotic remodeling
Immune cells	Anti-inflammatory modulation	Immunosuppressive tumor microenvironment

**Table 2:** Translational Landscape of NRF2 Modulation in Dermatological Disorders.

Compound	NRF2 Activation Mechanism	Main Dermatologic Indications	Route of Administration	Clinical/Development Status
Tapinarof (Stilbenoid; AhR agonist)	Indirect NRF2 activation via AhR–NRF2 crosstalk	Psoriasis; Atopic dermatitis	Topical	Phase III; FDA-approved (psoriasis, AD)
Dimethyl fumarate (DMF) (α,β-unsaturated fumaric acid ester)	Covalent KEAP1 cysteine modification (Cys151)	Psoriasis	Oral	Approved (psoriasis; also approved for MS)
Indomethacin (Indole acetic acid NSAID)	Electrophilic KEAP1 modification; indirect NRF2 activation	Photodamage (experimental); inflammatory dermatoses	Topical/systemic	Approved (non-dermatologic use); dermatology evidence preclinical/small clinical studies
Simvastatin (HMG-CoA reductase inhibitor)	Redox-sensitive signaling modulation; indirect NRF2 activation	Vitiligo (repurposing attempts)	Oral	Phase II (negative dermatology results); approved (hyperlipidemia)
Folic acid (Vitamin; pteroylmonoglutamic acid)	Modulation of KEAP1 expression; NRF2 nuclear translocation (indirect)	Vitiligo (adjunct therapy)	Oral	Clinical adjunct studies; approved (nutritional supplement)
Omaveloxolone (Semisynthetic triterpenoid)	Electrophilic KEAP1 modification (Michael acceptor)	Radiation dermatitis	Topical/systemic	Phase II (dermatology); approved for Friedreich’s ataxia (systemic)
Sulforaphane (SFN) (Isothiocyanate)	Covalent KEAP1 cysteine modification (Cys151, Cys273, Cys288)	Photoaging; psoriasis models; AD models	Topical/oral	Phase I (dermatology); Phase II (systemic antioxidant); investigational
Molecular hydrogen (H_2_) (Small molecule gas)	Indirect NRF2 stabilization via ROS modulation	Vitiligo (experimental); inflammatory dermatoses	Oral/inhaled/topical	Preclinical + early exploratory clinical studies; experimental
Bixin (Apocarotenoid)	Canonical KEAP1-dependent activation	Photodamage; hair graying (models)	Topical/systemic (models)	Preclinical (in vivo validated); experimental
Paeoniflorin (Monoterpene glycoside)	Disruption of KEAP1–NRF2 complex; PI3K/Akt modulation (indirect)	UVA damage; inflammatory epithelial injury	Oral/topical (experimental)	Preclinical; experimental
Baicalein (Flavone; polyphenol)	NRF2 nuclear translocation; possible KEAP1 interaction (direct binding not demonstrated)	Vitiligo models; AD models; UV injury	Topical/oral (experimental)	Preclinical; Phase I safety (non-derm); investigational (non-derm)
Berberine (Isoquinoline alkaloid)	NRF2/ARE activation; ROS reduction (indirect)	Vitiligo models	Topical/oral (experimental)	Preclinical; experimental
Apigenin (Flavonoid)	KEAP1 interference; ERK/PI3K-Akt modulation (indirect)	Vitiligo models; oxidative dermatoses	Topical/oral (experimental)	Preclinical; cosmetic/supplement use
Afzelin (Flavonol glycoside)	GSK-3β inhibition → NRF2 nuclear retention (indirect)	Vitiligo models	Experimental	Preclinical; experimental
6-Shogaol (Phenolic α,β-unsaturated ketone)	Electrophilic KEAP1 cysteine modification (proposed)	Vitiligo models	Experimental	Preclinical; experimental

## Data Availability

No new data were created or analyzed in this study.

## Abbreviations

AD	Atopic dermatitis
AhR	Aryl hydrocarbon receptor
ARE	Antioxidant response element
CBP	CREB-binding protein
COX	Cyclooxygenase
*CUL3*	Cullin 3
DAG	Diacetone-D-glucofuranose
DES	Deep eutectic solvents
DMF	Dimethyl fumarate
DNA	Deoxyribonucleic acid
DPPA	Diphenylphosphoryl azide
ERK	Extracellular signal-regulated kinase
*GCLC*	Glutamate–cysteine ligase catalytic subunit
GSK-3β	Glycogen synthase kinase 3 beta
HO-1	Heme oxygenase 1
H_2_	Molecular hydrogen
IL	Interleukin
JAK	Janus kinase
*KEAP1*	Kelch-like ECH-associated protein 1
MAPK	Mitogen-activated protein kinase
MITF	Microphthalmia-associated transcription factor
NF-κB	Nuclear factor kappa B
*NFE2L2*	Nuclear factor erythroid 2–related factor 2 gene
NLRP3	NOD-like receptor family pyrin domain-containing 3
NQO1	NAD(P)H quinone dehydrogenase 1
NRF2	Nuclear factor erythroid 2–related factor 2
NSAID	Non-steroidal anti-inflammatory drug
PAL	Phenylalanine ammonia-lyase
PF	Paeoniflorin
PI3K	Phosphoinositide 3-kinase
PKC	Protein kinase C
PUVA	Psoralen plus ultraviolet A
ROS	Reactive oxygen species
SCF	SKP1–Cullin–F-box
SFN	Sulforaphane
SLE	Systemic lupus erythematosus
TBAI	Tetrabutylammonium iodide
TGF-β	Transforming growth factor beta
UGT	UDP-glycosyltransferase
UV	Ultraviolet
VEGF	Vascular endothelial growth factor

## References

[1] Yamamoto M., Kensler T.W., Motohashi H. (2018). The KEAP1–NRF2 system: A thiol-based sensor–effector apparatus for maintaining redox homeostasis. Physiol. Rev..

[2] Itoh K., Wakabayashi N., Katoh Y., Ishii T., Igarashi K., Engel J.D., Yamamoto M. (1999). KEAP1 represses nuclear activation of antioxidant responsive elements by NRF2 through binding to the amino-terminal Neh2 domain. Genes Dev..

[3] Kobayashi A., Kang M.-I., Okawa H., Ohtsuji M., Zenke Y., Chiba T., Igarashi K., Yamamoto M. (2004). Oxidative stress sensor KEAP1 functions as an adaptor for CUL3-based e3 ligase to regulate proteasomal degradation of NRF2. Mol. Cell. Biol..

[4] Zhang D.D., Lo S.C., Cross J.V., Templeton D.J., Hanninke M. (2004). KEAP1 is a redox-regulated substrate adaptor protein for a CUL3-dependent ubiquitin ligase complex. Mol. Cell. Biol..

[5] Kahremany S., Hofmann L., Gruzman A., Dinkova-Kostova A.T., Cohen G. (2022). NRF2 in dermatological disorders: Pharmacological activation for protection against cutaneous photodamage and photodermatosis. Free Radic. Biol. Med..

[6] Kobayashi E.H., Suzuki T., Funayama R., Nagashima T., Hayashi M., Sekine H., Tanaka N., Moriguchi T., Motohashi H., Nakayama K. (2016). NRF2 suppresses macrophage inflammatory response by blocking proinflammatory cytokine transcription. Nat. Commun..

[7] Dinkova-Kostova A.T., Copple I.M. (2023). Advances and challenges in therapeutic targeting of NRF2. Trends Pharmacol. Sci..

[8] DeNicola G.M., Karreth F.A., Humpton T.J., Gopinathan A., Wei C., Frese K., Mangal D., Yu K.H., Yeo C.J., Calhoun E.S. (2011). Oncogene-induced NRF2 transcription promotes ROS detoxification and tumorigenesis. Nature.

[9] Wang H., Liu K., Geng M., Gao P., Wu X., Hai Y., Li Y., Li Y., Luo L., Hayes J.D. (2013). RXRα inhibits the NRF2–ARE signaling pathway through a direct interaction with the neh7 domain of NRF2. Cancer Res..

[10] Wakabayashi N., Dinkova-Kostova A.T., Holtzclaw W.D., Kang M.-I., Kobayashi A., Yamamoto M., Kensler T.W., Talalay P. (2004). Protection against electrophile and oxidant stress by induction of the phase 2 response: Fate of cysteines of the keap1 sensor modified by inducers. Proc. Natl. Acad. Sci. USA.

[11] Ichimura Y., Waguri S., Sou Y.-S., Kageyama S., Hasegawa J., Ishimura R., Saito T., Yang Y., Kouno T., Fukutomi T. (2013). Phosphorylation of p62 activates the KEAP1-NRF2 pathway during selective autophagy. Mol. Cell..

[12] Salazar M., Rojo A.I., Velasco D., de Sagarra R.M., Cuadrado A. (2006). Glycogen synthase kinase-3β inhibits the xenobiotic and antioxidant cell response by direct phosphorylation and nuclear exclusion of the transcription factor NRF2. J. Biol. Chem..

[13] Cuadrado A., Martín-Moldes Z., Ye J., Lastres-Becker I. (2014). Transcription factors NRF2 and NF-κB are coordinated effectors of the Rho family, GTP-binding protein RAC1 during Inflammation. J. Biol. Chem..

[14] Wakabayashi N., Slocum S.L., Skoko J.J., Shin S., Kensler T.W. (2010). When NRF2 talks, who’s listening?. Antioxid. Redox Signal..

[15] Ma C., Gu C., Lian P., Wazir J., Lu R., Ruan B., Wei L., Li L., Pu W., Peng Z. (2023). Sulforaphane alleviates psoriasis by enhancing antioxidant defense through KEAP1-NRF2 pathway activation and attenuating inflammatory signaling. Cell Death Dis..

[16] Koch M., Kockmann T., Rodriguez E., Wehkamp U., Hiebert P., Greenwald M.B.-Y., Stölzl D., Beer H.-D., Tschachler E., Weidinger S. (2023). Quantitative proteomics identifies reduced NRF2 activity and mitochondrial dysfunction in atopic dermatitis. J. Investig. Dermatol..

[17] Helou D.G., Noël B., Gaudin F., Groux H., El Ali Z., Pallardy M., Chollet-Martin S., Kerdine-Römer S. (2019). Cutting edge: NRF2 regulates neutrophil recruitment and accumulation in skin during contact hypersensitivity. J. Immunol..

[18] Jian Z., Li K., Song P., Zhu G., Zhu L., Cui T., Liu B., Tang L., Wang X., Wang G. (2011). Heme oxygenase-1 protects human melanocytes from H_2_O_2_-induced oxidative stress via the NRF2-ARE pathway. J. Investig. Dermatol..

[19] Song P., Li K., Liu L., Wang X., Jian Z., Zhang W., Wang G., Li C., Gao T. (2016). Genetic polymorphism of the NRF2 promoter region is associated with vitiligo risk in han chinese populations. J. Cell. Mol. Med..

[20] Rada P., Rojo A.I., Chowdhry S., McMahon M., Hayes J.D., Cuadrado A. (2011). SCF/β-TrCP promotes glycogen synthase kinase 3-dependent degradation of the NRF2 transcription factor in a KEAP1-independent manner. Mol. Cell. Biol..

[21] Talalay P., Fahey J.W., Healy Z.R., Wehage S.L., Benedict A.L., Min C., Dinkova-Kostova A.T. (2007). Sulforaphane mobilizes cellular defenses that protect skin against damage by UV radiation. Proc. Natl. Acad. Sci. USA.

[22] Tao S., Park S.L., Rojo de la Vega M., Zhang D.D., Wondrak G.T. (2015). Systemic administration of the apocarotenoid bixin protects skin against solar UV-induced damage through activation of NRF2. Free Radic. Biol. Med..

[23] Rojo de la Vega M., Zhang D.D., Wondrak G.T. (2018). Topical bixin confers NRF2-dependent protection against photodamage and hair graying in mouse skin. Front. Pharmacol..

[24] Kubben N., Zhang W., Wang L., Voss T.C., Yang J., Qu J., Liu G.H., Misteli T. (2016). Repression of the antioxidant NRF2 pathway in premature aging. Cell.

[25] National Institutes of Helath (2014). RTA-408 Lotion in Patients at Risk for Radiation Dermatitis (PRIMROSE).

[26] Nakagami Y. (2017). NRF2 activators as therapy for acute radiation dermatitis. J. Rare Dis. Res. Treat..

[27] Braun S., Hanselmann C., Gassmann M.G., auf dem Keller U., Born-Berclaz C., Chan K., Kan Y.W., Werner S. (2002). NRF2 transcription factor, a novel target of keratinocyte growth factor action which regulates gene expression and accelerates cutaneous wound healing. Mol. Cell. Biol..

[28] Li Y., Ma F., Li H., Song Y., Zhang H., Jiang Z., Wu H. (2018). Dimethyl fumarate accelerates wound healing under diabetic condition. J. Mol. Endocrinol..

[29] Li M., Yu H., Pan H., Zhou X., Ruan Q., Kong D., Chu Z., Li H., Huang J., Huang X. (2019). NRF2 suppression delays diabetic wound healing through sustained oxidative stress and inflammation. Front. Pharmacol..

[30] Barakat M., Han C., Chen L., David B.P., Shi J., Xu A., Skowron K.J., Johnson T., Woods R.A., Ankireddy A. (2024). Non-electrophilic NRF2 activators promote wound healing in human keratinocytes and diabetic mice and demonstrate selective downstream gene targeting. Sci. Rep..

[31] Kavian N., Servettaz A., Weill B., Batteux F. (2018). The NRF2–antioxidant response element signaling pathway controls fibrosis and autoimmunity in scleroderma. Front. Immunol..

[32] Wu R., Zhang H., Zhao M., Li J., Hu Y., Fu J., Pi J., Wang H., Xu Y. (2020). NRF2 in keratinocytes protects against skin fibrosis via regulating epidermal lesion and inflammatory response. Biochem. Pharmacol..

[33] Knatko E.V., Ibbotson S.H., Zhang Y., Higgins M., Fahey J.W., Talalay P., Dawe R.S., Ferguson J., Huang J.T.-J., Clarke R. (2015). NRF2 activation protects against solar-simulated ultraviolet radiation in mice and humans. Cancer Prev. Res..

[34] Xu C., Huang M.-T., Shen G., Yuan X., Lin W., Khor T.O., Conney A.H., Kong A.-N.T. (2006). Inhibition of 7,12-dimethylbenz(a)anthracene-induced skin tumorigenesis in C57BL/6 Mice by sulforaphane is mediated by nuclear factor E2-related factor 2. Cancer Res..

[35] Schäfer M., Dütsch S., auf dem Keller U., Navid F., Schwarz A., Johnson D.A., Johnson J.A., Werner S. (2010). NRF2 establishes a glutathione-mediated gradient of UVB cytoprotection in the epidermis. Genes Dev..

[36] auf dem Keller U., Huber M., Beyer T.A., Kumin A., Siemes C., Braun S., Bugnon P., Mitropoulos V., Johnson D.A., Johnson J.A. (2006). NRF transcription factors in keratinocytes are essential for skin tumor prevention but not for wound healing. Mol. Cell. Biol..

[37] Shibata T., Ohta T., Tong K.I., Kokubu A., Odogawa R., Tsuta K., Asamura H., Yamamoto M., Hirohashi S. (2008). Cancer related mutations in NRF2 impair its recognition by KEAP1-CUL3 E3 ligase and promote malignancy. Proc. Natl. Acad. Sci. USA.

[38] Aly D.G., Shahin R.S. (2010). Oxidative stress in lichen planus. Acta Dermatovenerol. APA.

[39] Sagdic A., Sener O., Bulucu F., Karadurmus N., Yamanel L., Tasci C., Naharci I., Ocal R., Aydin A. (2011). Oxidative stress status in patients with chronic idiopathic urticaria. Allergol. Immunopathol..

[40] Matusiak Ł., Szczęch J., Bieniek A., Nowicka-Suszko D., Szepietowski J.C. (2017). Increased interleukin (IL)-17 serum levels in patients with hidradenitis suppurativa: Implications for treatment with anti-IL-17 agents. J. Am. Acad. Dermatol..

[41] Baird L., Yamamoto M. (2020). The molecular mechanisms regulating the KEAP1–NRF2 pathway. Mol. Cell. Biol..

[42] Schäfer M., Farwanah H., Willrodt A.-H., Huebner A.J., Sandhoff K., Roop D., Hohl D., Bloch W., Werner S. (2012). NRF2 links epidermal barrier function with antioxidant defense. EMBO Mol. Med..

[43] Rojo de la Vega M., Chapman E., Zhang D.D. (2018). NRF2 and the hallmarks of cancer. Cancer Cell.

[44] Schäfer M., Werner S. (2015). NRF2—A regulator of keratinocyte redox signaling. Free Radic. Biol. Med..

[45] Gęgotek A., Skrzydlewska E. (2015). The role of transcription factor NRF2 in skin cells metabolism. Arch. Dermatol. Res..

[46] Liu X.P., Goldring C.E.P., Copple I.M., Wang H.Y., Wei W., Kitteringham N.R., Park B.K. (2007). Extract of ginkgo biloba induces phase 2 genes through KEAP1–NRF2–ARE signaling pathway. Life Sci..

[47] Zhang S., Yi X., Su X., Jian Z., Cui T., Guo S., Gao T., Li C., Li S., Xiao Q. (2019). Ginkgo biloba extract protects human melanocytes from H_2_O_2_-induced oxidative stress by activating NRF2. J. Cell. Mol. Med..

[48] Chen C.C., Chiang A.N., Liu H.N., Chang Y.T. (2014). EGb-761 prevents ultraviolet B-induced photoaging via inactivation of mitogen-activated protein kinases and proinflammatory cytokine expression. J. Dermatol. Sci..

[49] Parsad D., Pandhi R., Juneja A. (2003). Effectiveness of oral ginkgo biloba in treating limited, slowly spreading vitiligo. Clin. Exp. Dermatol..

[50] Szczurko O., Shear N.H., Taddio A., Boon H. (2011). Ginkgo biloba for the treatment of vitiligo vulgaris: An open-label pilot clinical trial. BMC Complement. Altern. Med..

[51] Smith S.H., Jayawickreme C., Rickard D.J., Nicodeme E., Bui T., Simmons C., Coquery C.M., Neil J., Pryor W.M., Mayhew D. (2017). Tapinarof is a natural AhR agonist that resolves skin inflammation in mice and humans. J. Investig. Dermatol..

[52] Zhang Y., Du M., Yu Y.-F., Xu S.-X., Zhao S.-C., Wang H.-T. (2014). Synthesis of (E)-3,5-dihydroxy-4-isopropylstilbene under microwave irradiation. J. Chem. Pharm. Res..

[53] Andrews I.P., Calandra N., Davis T.A., Sudini R.R. (2020). Process for Preparing Tapinarof. U.S. Patent.

[54] Lin C.-H., Ko H.-H., Wu J.-Y., Chang H.-S., Yen C.-H., Chiu C.-C., Chen Y.-F. (2025). Dual activation of AhR and NRF2 pathways by the natural stilbenoid tapinarof protects against particulate matter-induced skin barrier dysfunction. Toxicol. Appl. Pharmacol..

[55] Silverberg J.I., Boguniewicz M., Quintana F.J., Clark R.A., Gross L., Hirano I., Tallman A.M., Brown P.M., Fredericks D., Rubenstein D.S. (2024). Tapinarof validates the aryl hydrocarbon receptor as a therapeutic target: A clinical review. J. Allergy Clin. Immunol..

[56] Lebwohl M.G., Stein Gold L., Strober B., Papp K.A., Armstrong A.W., Bagel J., Kircik L., Ehst B., Hong C.-H.H., Soung J. (2021). Phase 3 trials of tapinarof cream for plaque psoriasis. N. Engl. J. Med..

[57] U.S. Food and Drug Administration (2024). VTAMA (Tapinarof) Cream, 1%: Prescribing Information.

[58] Dedè F., Piccolo O., Vigo D. (2021). Dimethyl fumarate: Heterogeneous catalysis for the development of an innovative flow synthesis. Org. Process Res. Dev..

[59] Lima M.T., Finelli F.G., de Oliveira A.V.B., Kartnaller V., Cajaiba J.F., Leão R.A.C., de Souza R.O.M.A. (2020). Continuous-flow synthesis of dimethyl fumarate: A powerful small molecule for the treatment of psoriasis and multiple sclerosis. RSC Adv..

[60] Linker R.A., Lee D.-H., Ryan S., van Dam A.M., Conrad R., Bista P., Zeng W., Hronowsky X., Buko A., Chollate S. (2011). Fumaric acid esters exert neuroprotective effects in neuroinflammation via activation of the NRF2 antioxidant pathway. Brain.

[61] Mrowietz U., Szepietowski J.C., Loewe R., van de Kerkhof P., Lamarca J., Ocker W.G., Tebbs V.M., Pau-Charles I. (2017). Efficacy and safety of LAS41008 (dimethyl fumarate) in adults with moderate-to-severe chronic plaque psoriasis: A randomized, double-blind, Fumaderm^®^- and placebo-controlled trial (BRIDGE). Br. J. Dermatol..

[62] Leh Lehmann J.C.U., Listopad J.J., Rentzsch C.U., Igney F.H., von Bonin A., Hennekes H.H., Asadullah K., Docke W.-D.F. (2007). Dimethyl fumarate induces immunosuppression via glutathione depletion and subsequent induction of heme oxygenase 1. J. Investig. Dermatol..

[63] Magedov V., Maklakov S.A., Smushkevich Y.I. (2005). New procedure for obtaining indomethacin. Chem. Heterocycl. Compd..

[64] Eisenstein A., Hilliard B.K., Pope S.D., Zhang C., Taskar P., Waizman D.A., Israni-Winger K., Tian H., Luan H.H., Wang A. (2022). Activation of the transcription factor NRF2 mediates the anti-inflammatory properties of a subset of over-the-counter and prescription NSAIDs. Immunity.

[65] Farr P.M., Diffey B.L. (1986). A quantitative study of the effect of topical indomethacin on cutaneous erythema induced by UVB and UVC radiation. Br. J. Dermatol..

[66] Alberts A.W. (1988). Discovery, biochemistry and biology of lovastatin. Am. J. Cardiol..

[67] Endo A. (2010). A historical perspective on the discovery of statins. Proc. Jpn. Acad. Ser. B.

[68] Gao X., Xie X., Pashkov I., Sawaya M.R., Laidman J., Zhang W., Cacho R., Yeates T.O., Tang Y. (2009). Directed evolution and structural characterization of a simvastatin synthase. Chem. Biol..

[69] Liao J.K., Laufs U. (2005). Pleiotropic effects of statins. Annu. Rev. Pharmacol. Toxicol..

[70] Chang Y., Li S., Guo W., Yang Y., Zhang W., Zhang Q., He Y., Yi X., Cui T., An Y. (2017). Simvastatin protects human melanocytes from H_2_O_2_-induced oxidative stress by activating NRF2. J. Investig. Dermatol..

[71] Vanderweil S.G., Amano S., Ko W.-C., Richmond J.M., Kelley M., Makredes Senna M., Pearson A., Chowdary S., Hartigan C., Barton B. (2017). A double-blind, placebo-controlled, phase-II clinical trial to evaluate oral simvastatin as a treatment for vitiligo. J. Am. Acad. Dermatol..

[72] Wehrli C. (1995). Method for the Production of Folic Acid Using a Novel Diimine Intermediate. U.S. Patent.

[73] Wang G., Zhang J., Wang S. (2016). Folic Acid Synthesis Method. China Patent.

[74] Serrano-Amatriain C., Ledesma-Amaro R., López-Nicolás R., Ros G., Jiménez A., Revuelta J.L. (2016). Folic acid production by engineered ashbya gossypii. Metab. Eng..

[75] Du P., Zhang S., Li S., Yang Y., Kang P., Chen J., Gao T., Li C., Zhang Q., Zhang W. (2021). Folic acid protects melanocytes from oxidative stress via activation of NRF2 and inhibition of HMGB1. Oxidative Med. Cell. Longev..

[76] Juhlin L., Olsson M.J. (1997). Improvement of vitiligo after oral treatment with vitamin B12 and folic acid and the importance of sun exposure. Acta Derm. Venereol..

[77] Anderson E., Decker A., Liu X. (2015). 2,2-Difluoropropionamide Derivatives of Bardoxolone Methyl, Polymorphic Forms and Methods of Use Thereof. U.S. Patent.

[78] Reisman S.A., Lee C.Y.I., Meyer C.J., Proksch J.W., Sonis S.T., Ward K.W. (2014). Topical application of the synthetic triterpenoid RTA 408 protects mice from radiation-induced dermatitis. Radiat. Res..

[79] Reisman S.A., Goldsberry A.R., Lee C.-Y.I., O’Grady M.L., Proksch J.W., Ward K.W., Meyer C.J. (2015). Topical application of RTA 408 lotion activates NRF2 in human skin and is well-tolerated by healthy human volunteers. BMC Dermatol..

[80] U.S. Food and Drug Administration (2023). Skyclarys (Omaveloxolone) Prescribing Information.

[81] Zhang Y., Talalay P., Cho C.G., Posner G.H. (1992). A major inducer of anticarcinogenic protective enzymes from broccoli: Isolation and elucidation of structure. Proc. Natl. Acad. Sci. USA.

[82] Fahey J.W., Zalcmann A.T., Talalay P. (2001). The chemical diversity and distribution of glucosinolates and isothiocyanates among plants. Phytochemistry.

[83] Dinkova-Kostova A.T., Kostov R.V. (2012). Glucosinolates and isothiocyanates in health and disease. Trends Mol. Med..

[84] De Nicola G.R., Rollin P., Mazzon E., Iori R. (2014). Novel gram-scale production of enantiopure *R*-sulforaphane from Tuscan black kale seeds. Molecules.

[85] Vermeulen M., Zwanenburg B., Chittenden G.J.F., Verhagen H. (2003). Synthesis of isothiocyanate-derived mercapturic acids. Eur. J. Med. Chem..

[86] Conaway C.C., Wang C.-X., Pittman B., Yang Y.-M., Schwartz J.E., Tian D., McIntee E.J., Hecht S.S., Chung F.-L. (2005). Phenethyl isothiocyanate and sulforaphane and their n-acetylcysteine conjugates inhibit malignant progression of lung adenomas induced by tobacco carcinogens in A/J mice. Cancer Res..

[87] Chen X., Li Z., Sun X., Ma H., Chenc X., Rena J., Hu K. (2011). New method for the synthesis of sulforaphane and related isothiocyanates. Synthesis.

[88] Whitesell J.K., Wong M.S. (1994). Asymmetric synthesis of chiral sulfinate esters and sulfoxides. Synthesis of sulforaphane. J. Org. Chem..

[89] Fernández I., Khiar N., Llera J.M., Alcudia F. (1992). Asymmetric synthesis of alkane- and arenesulfinates of diacetone-D-glucose (DAG): An improved and general route to both enantiomerically pure sulfoxides. J. Org. Chem..

[90] Khiar N., Werner S., Mallouk S., Lieder F., Alcudia A., Fernández I. (2009). Enantiopure sulforaphane analogues with various substituents at the sulfinyl sulfur: Asymmetric synthesis and biological activities. J. Org. Chem..

[91] Alcarranza M., Villegas I., Muñoz-García R., Recio R., Fernández I., Alarcón-de-la-Lastra C. (2022). Immunomodulatory effects of (*R*)-sulforaphane on LPS-activated murine immune cells: Molecular signaling pathways and epigenetic changes in histone markers. Pharmaceuticals.

[92] Elhalem E., Recio R., Werner S., Lieder F., Calderón Montaño J.M., López Lázaro M., Fernández I., Khiar N. (2014). Sulforaphane homologues: Enantiodivergent synthesis of both enantiomers, activation of the NRF2 transcription factor and selective cytotoxic activity. Eur. J. Med. Chem..

[93] Alcarranza M., Alarcón-de-la-Lastra C., Recio Jiménez R., Fernández I., Castejón Martínez M.L., Villegas I. (2024). Immunomodulatory effects and regulatory mechanisms of (R)-6-HITC, an isothiocyanate from wasabi (*Eutrema japonicum*), in an ex vivo mouse model of LPS-induced inflammation. J. Agric. Food Chem..

[94] Alcarranza M., Villegas I., Recio R., Muñoz-García R., Fernández I., Alarcón-de-la-Lastra C. (2023). (*R*)-8-Methylsulfinyloctyl isothiocyanate from Nasturtium officinale inhibits LPS-induced immunoinflammatory responses in mouse peritoneal macrophages: Chemical synthesis and molecular signaling pathways involved. Food Funct..

[95] Schenk W.A., Dürr M. (1997). Synthesis of (*R*)-sulforaphane using [CpRu((R,R)-CHIRAPHOS)]^+^ as chiral auxiliary. Chem. Eur. J..

[96] Posner G.H., Cho C.G., Green J.V., Zhang Y., Talalay P. (1994). Design and synthesis of bifunctional isothiocyanate analogs of sulforaphane: Correlation between structure and potency as inducers of anticarcinogenic detoxication enzymes. J. Med. Chem..

[97] Janczewski Ł. (2022). Sulforaphane and Its bifunctional analogs: Synthesis and biological activity. Molecules.

[98] Kiełbasiński P., Łuczak J., Cierpiał T., Błaszczyk J., Sieroń L., Wiktorska K., Lubelska K., Milczarek M., Chilmończyk Z. (2014). New enantiomeric fluorine-containing derivatives of sulforaphane: Synthesis, absolute configurations and biological activity. Eur. J. Med. Chem..

[99] Cierpiał T., Kiełbasiński P., Kwiatkowska M., Łyżwa P., Lubelska K., Kuran D., Dąbrowska A., Kruszewska H., Mielczarek L., Chilmończyk Z. (2020). Fluoroaryl analogs of sulforaphane—A group of compounds of anticancer and antimicrobial activity. Bioorg. Chem..

[100] Fortunato S., Lenzi C., Granchi C., Citi V., Martelli A., Calderone V., Di Pietro S., Signore G., Di Bussolo V., Minutolo F. (2019). First examples of H_2_S-releasing glycoconjugates: Stereoselective synthesis and anticancer activities. Bioconjug. Chem..

[101] Prieto L.A., Khiar-Fernandez N., Calderon-Montaño J.M., Lopez-Lázaro M., Lucía-Tamudo J., Nogueira J.J., Leon R., Moreno N., Valdivia V., Recio R. (2025). Exploring the broad-spectrum activity of carbohydrate-based iberin analogues: From anticancer effect to antioxidant properties. Eur. J. Med. Chem..

[102] Psurski M., Janczewski Ł., Świtalska M., Gajda A., Goszczyński T.M., Oleksyszyn J., Wietrzyk J., Gajda T. (2017). Novel phosphonate analogs of sulforaphane: Synthesis, in vitro and in vivo activity. Eur. J. Med. Chem..

[103] Khiar El Wahabi N., Fernández Fernández I., Recio Jiménez R. (2015). Sulforaphane-Derived Compounds, Production Method Thereof and the Medical, Food and Cosmetic Use of Same. U.S. Patent.

[104] Johns Hopkins University (2019). Effect of Topical Sulforaphane on Skin Aging and with Ultraviolet and Visible Light Exposure.

[105] Sidney Kimmel Comprehensive Cancer Center at Johns Hopkins (2018). Study to Evaluate the Effect of Sulforaphane in Broccoli Sprout Extract on Breast Tissue.

[106] Wu W., Peng G., Yang F., Zhang Y., Mu Z., Han X. (2019). Sulforaphane has a therapeutic effect in an atopic dermatitis murine model and activates the NRF2/HO-1 axis. Mol. Med. Rep..

[107] Ohta S. (2014). Molecular hydrogen as a preventive and therapeutic medical gas: Initiation, development and potential of hydrogen medicine. Pharmacol. Ther..

[108] Fang W., Tang L., Wang G., Lin J., Liao W., Pan W., Xu J. (2020). Molecular hydrogen protects human melanocytes from oxidative stress by activating NRF2 signaling. J. Investig. Dermatol..

[109] Kato S., Saitoh Y., Iwai K., Miwa N. (2012). Hydrogen-rich electrolyzed warm water represses wrinkle formation against UVA ray together with type-I collagen production and oxidative-stress diminishment in fibroblasts and cell-injury prevention in keratinocytes. J. Photochem. Photobiol. B Biol..

[110] Ohno K., Ito M., Ichihara M., Ito M. (2012). Molecular hydrogen as an emerging therapeutic medical gas for neurodegenerative and other diseases. Oxidative Med. Cell. Longev..

[111] Scotter M. (2009). The chemistry and analysis of annatto food colouring: A review. Food Addit. Contam. Part A.

[112] Rojo de la Vega M., Krajisnik A., Zhang D.D., Wondrak G.T. (2017). Targeting NRF2 for Improved Skin Barrier Function and Photoprotection: Focus on the Achiote-Derived Apocarotenoid Bixin. Nutrients.

[113] Lu B., An F., Cao L., Gao Q., Wang X., Yang Y., Liu P., Yang B., Chen T., Li X.-C. (2020). Comparative transcriptomics characterized the distinct biosynthetic abilities of terpenoid and paeoniflorin biosynthesis in herbaceous peony strains. PeerJ.

[114] Boo Y.C. (2020). Natural NRF2 modulators for skin protection. Antioxidants.

[115] Xiang Y., Zhang Q., Wei S., Huang C., Li Z., Gao Y. (2020). Paeoniflorin: A monoterpene glycoside from plants of the paeoniaceae family with diverse anticancer activities. J. Pharm. Pharmacol..

[116] Wen J., Sun H., Zhang G., Li H., Ren H., Li X., Du K., Changet Y. (2025). Green microextraction using deep eutectic solvents: Computer-aided analysis of bioactive components in natural products. Ind. Crops. Prod..

[117] Xu P., Li Q., Liang W., Hu Y., Chen R., Lou K., Zhan L., Wu X., Pu J. (2023). A tissue-specific profile of miRNAs and their targets related to paeoniaflorin and monoterpenoids biosynthesis in Paeonia lactiflora Pall. by transcriptome, small RNAs and degradome sequencing. PLoS ONE.

[118] Ye Y., Pei H., Cao X., Liu X., Li Z., Wang B., Pan Y., Zheng J. (2023). The study of a novel paeoniflorin-converting enzyme from Cunninghamella blakesleeana. Molecules.

[119] Ni J., Yang M., Zheng X., Wang M., Xiao Q., Han H., Dong P. (2024). Synthesis, antioxidant activity, and molecular docking of novel paeoniflorin derivatives. Chem. Biol. Drug Des..

[120] Wu X.X., Huang X.L., Chen R.R., Li T., Ye H.J., Xie W., Huang Z.M., Cao G.Z. (2019). Paeoniflorin prevents intestinal barrier disruption and inhibits lipopolysaccharide (LPS)-induced inflammation in Caco-2 cell monolayers. Inflammation.

[121] Lu Y.-S., Jiang Y., Yuan J.-P., Jiang S.-B., Yang Y., Zhu P.-Y., Sun Y.-Z., Qi R.-Q., Liu T., Wang H.-X. (2020). UVA induced oxidative stress was inhibited by paeoniflorin/NRF2 signaling or PLIN2. Front. Pharmacol..

[122] Jiang T., Liu X., Wang S., Chen Y., Wang Y., Li X., Yao G. (2025). Paeoniflorin alleviated experimental Sjögren’s syndrome by inhibiting NLRP3 inflammasome activation of submandibular gland cells via activating NRF2/HO-1 pathway. Free Radic. Biol. Med..

[123] Sun X., Wang X., Zhao Z., Chen J., Li C., Zhao G. (2021). Paeoniflorin inhibited nod-like receptor protein-3 inflammasome and NF-κB-mediated inflammatory reactions in diabetic foot ulcer by inhibiting the chemokine receptor CXCR2. Drug Dev. Res..

[124] Liu X., Li X., Li X., Li Z., Zhao D., Liu S., Zhang M., Zhang F., Zhu P., Chen J. (2019). The efficacy and safety of total glucosides of peony in the treatment of primary Sjögren’s syndrome: A multi-center, randomized, double-blinded, placebo-controlled clinical trial. Clin. Rheumatol..

[125] Chen Z., Li X.-P., Li Z.-J., Xu L., Li X.-M. (2013). Reduced hepatotoxicity by total glucosides of paeony in combination treatment with leflunomide and methotrexate for patients with active rheumatoid arthritis. Int. Immunopharmacol..

[126] Xiang N., Li X.-M., Zhang M.-J., Zhao D.-B., Zhu P., Zuo X.-X., Yang M., Su Y., Li Z.-G., Chen Z. (2015). Total glucosides of paeony can reduce the hepatotoxicity caused by methotrexate and leflunomide combination treatment of active rheumatoid arthritis. Int. Immunopharmacol..

[127] Wang Z.L., Wang S., Kuang Y., Hu Z.M., Qiao X., Ye M. (2018). A comprehensive review on phytochemistry, pharmacology, and flavonoid biosynthesis of Scutellaria baicalensis. Pharm. Biol..

[128] Shang X., He X., He X., Li M., Zhang R., Fan P., Zhang Q., Jia Z. (2010). The genus Scutellaria: An ethnopharmacological and phytochemical review. J. Ethnopharmacol..

[129] Nik Salleh N.N.H., Othman F.A., Kamarudin N.A., Tan S.C. (2020). The biological activities and therapeutic potentials of baicalein extracted from Oroxylum indicum: A systematic review. Molecules.

[130] Fujita M., Shiota S., Kuroda T., Hatano T., Yoshida T., Mizushima T., Tsuchiya T. (2005). Remarkable synergies between baicalein and tetracyclie, and baicalein and β-lactams against methicillin-resistant Staphylococcus aureus. Microbiol. Immunol..

[131] Kim S., Sohn D.W., Kim Y.C., Kim S.A., Lee S.K., Kim H.S. (2007). Fine tuning of a reported synthetic route for biologically active flavonoid, baicalein. Arch. Pharm. Res..

[132] Chen D.Z., Yang J., Yang B., Wu Y.S., Wu T. (2010). Total synthesis of baicalein. J. Asian Nat. Prod. Res..

[133] Ma J., Li S., Zhu L., Guo S., Yi X., Cui T., He Y., Chang Y., Liu B., Li C. (2018). Baicalein protects human vitiligo melanocytes from oxidative stress through activation of NF-E2-related factor 2 (NRF2) signaling pathway. Free Radic. Biol. Med..

[134] Chi F., Cheng C., Liu K., Sun T., Zhang M., Hou Y., Bai G. (2025). Baicalein disrupts the KEAP1-NRF2 interaction to alleviate oxidative stress injury by inhibiting M1 macrophage polarization. Free Radic. Biol. Med..

[135] Yun M.-Y., Yang J.-H., Kim D.-K., Cheong K.-J., Song H.-H., Kim D.-H., Cheong K.-J., Kim Y.-I., Shin S.-C. (2010). Therapeutic effects of baicalein on atopic dermatitis-like skin lesions of NC/Nga mice induced by Dermatophagoides pteronyssinus. Int. Immunopharmacol..

[136] Kimura Y., Sumiyoshi M. (2011). Effects of baicalein and wogonin isolated from Scutellaria baicalensis roots on skin damage in acute UVB-irradiated hairless mice. Eur. J. Pharmacol..

[137] Li L., Gao H., Lou K., Luo H., Hao S., Yuan J., Liu Z., Dong R. (2021). Safety, tolerability, and pharmacokinetics of oral baicalein tablets in healthy Chinese subjects: A single-center, randomized, double-blind, placebo-controlled multiple-ascending-dose study. Clin. Transl. Sci..

[138] CSPC ZhongQi Pharmaceutical Technology Co., Ltd (2019). A Randomized, Double-Blind, Placebo-Controlled, Multicenter and Phase IIa Clinical Trial for the Effectiveness and Safety of Baicalein Tablets in the Treatment of Improve Other Aspects of Healthy Adult with Influenza Fever.

[139] Neag M.A., Mocan A., Echeverría J., Pop R.M., Bocsan C.I., Crișan G., Buzoianu A.D. (2018). Berberine: Botanical occurrence, traditional uses, extraction methods, and relevance in cardiovascular, metabolic, hepatic, and renal disorders. Front. Pharmacol..

[140] Tajiri M., Yamada R., Hotsumi M., Makabe K., Konno H. (2021). The total synthesis of berberine and selected analogues, and their evaluation as amyloid β aggregation inhibitors. Eur. J. Med. Chem..

[141] Yan X., Zheng J., Li W.-D.Z. (2023). Concise total syntheses of berberine and its analogues in multigram scale enabled by trifluoroacetic anhydride–promoted decarbonylative-elimination reaction. Tetrahedron Lett..

[142] Jiang W., Li S., Chen X., Zhang W., Chang Y., He Y., Zhang S., Su X., Gao T., Li C. (2019). Berberine protects immortalized line of human melanocytes from H_2_O_2_ induced oxidative stress via activation of NRF2 and Mitf signaling pathway. J. Dermatol. Sci..

[143] Elhalmoushy P.M., Elsheikh M.A., Matar N.A., El Hadidy W.F., Kamel M.A., Omran G.A., Elnaggar Y.S.R. (2022). Novel berberine loaded hyalurosomes as a promising nanodermatological treatment for vitiligo: Biochemical, biological and gene expression studies. Int. J. Pharm..

[144] Zhou X., Wang F., Zhou R., Song X., Xie M. (2017). Apigenin: A current review on its beneficial biological activities. J. Food Biochem..

[145] Kumar S., Pandey A.K. (2013). Chemistry and Biological Activities of Flavonoids: An Overview. Sci. World J..

[146] Chemat F., Vian M.A., Cravotto G. (2012). Green extraction of natural products: Concept and principles. Int. J. Mol. Sci..

[147] Dai J., Mumper R.J. (2010). Plant Phenolics: Extraction, analysis and their antioxidant and anticancer properties. Molecules.

[148] Tang Q.-Q., Wang Z.-D., An X.-H., Zhou X.-Y., Zhang R.-Z., Zhan X., Zhang W., Zhou J. (2024). Apigenin ameliorates H_2_O_2_-induced oxidative damage in melanocytes through nuclear factor-E2-related factor 2 (NRF2) and phosphatidylinositol 3-kinase (PI3K)/protein kinase B (Akt)/mammalian target of rapamycin (mTOR) pathways and reducing the generation of reactive oxygen species (ROS) in zebrafish. Pharmaceuticals.

[149] Lu J., Zhou H., Hu J., Zhang R., Meng Z., Guan S. (2024). Apigenin reduces lipid droplet accumulation in hepatocytes by enhancing chaperone-mediated autophagy via AMPK. J. Agric. Food Chem..

[150] Zhang L., Li H., Xu S., Wen H., Yu C. (2025). Apigenin attenuates ischemia–reperfusion-induced pulmonary ferroptosis and fibrosis by activating the NRF2/HO-1/GPX4 axis in mice. Turk. J. Biol..

[151] Mohammadkhanizadeh A., Sheibani M., Taherkhani S., Nourabadi D., Mohamadi-Zarch S.M., Nikbakht F., Azizi Y. (2025). Protective effects of apigenin in neurodegeneration: An update on the potential mechanisms. Brain Disord..

[152] Hassan H., Delva C., Hunter C., Radparvar A., Miner K., Reimer H., Frasier K. (2025). Harnessing flavonoids and nutraceuticals for vitiligo via targeting oxidative stress and neural–melanocyte crosstalk. ARC J. Dermatol..

[153] Zhang B., Wang J., Zhao G., Lin M., Lang Y., Zhang D., Feng D., Tu C. (2020). Apigenin protects human melanocytes against oxidative damage by activation of the NRF2 pathway. Cell Stress Chaperones.

[154] Hostetler G.L., Ralston R.A., Schwartz S.J. (2017). Flavones: Food sources, bioavailability, metabolism, and bioactivity. Adv. Nutr..

[155] Zhang Z., ElSohly H.N., Li X.-C., Khan S.I., Broedel S.E., Raulli R.E., Cihlar R.L., Burandt C., Walker L.A. (2003). Phenolic compounds from Nymphaea odorata. J. Nat. Prod..

[156] Kim M., Shin S., Ryu D., Cho E., Yoo J., Park D., Jung E. (2021). Evaluating the sun protection factor of cosmetic formulations containing afzelin. Chem. Pharm. Bull..

[157] Boo Y.C. (2020). Emerging strategies to protect the skin from ultraviolet rays using plant-derived materials. Antioxidants.

[158] Gachon C.M.M., Langlois-Meurinne M., Saindrenan P. (2005). Plant secondary metabolism glycosyltransferases: The emerging functional analysis. Trends Plant Sci..

[159] Bowles D., Isayenkova J., Lim E.-K., Poppenberger B. (2005). Glycosyltransferases: Managers of small molecules. Curr. Opin. Plant Biol..

[160] Jung E., Kim J.H., Kim M.O., Choi J.H., Boo Y.C. (2017). Melanocyte-protective effect of afzelin is mediated by the NRF2–ARE signaling pathway via GSK-3β inactivation. Exp. Dermatol..

[161] Bhattarai S., Tran V.H., Duke C.C. (2001). The stability of gingerol and shogaol in aqueous solutions. J. Pharm. Sci..

[162] Suekawa M., Ishige A., Yuasa K., Sudo K., Aburada M., Hosoya E. (1984). Pharmacological studies on ginger. I. pharmacological actions of pungent constituents, (6)-gingerol and (6)-shogaol. J. Pharmacobiodyn..

[163] Kou X., Li X., Rahman M.R.T., Yan M., Huang H., Wang H., Su Y. (2017). Efficient dehydration of 6-gingerol to 6-shogaol catalyzed by an acidic ionic liquid under ultrasound irradiation. Food Chem..

[164] Kumar N.V., Murthy P.S., Manjunatha J.R., Bettadaiah B.K. (2014). Synthesis and quorum sensing inhibitory activity of key phenolic compounds of ginger and their derivatives. Food Chem..

[165] Mak K.-K., Zhang S., Sakirolla R., Balijepalli M.K., Dinkova-Kostova A.T., Epemolu O., Mohd Z., Pichika M.R. (2023). Synthesis of new shogaol analogues as NRF2 activators and evaluation of their anti-inflammatory activity, modes of action and metabolic stability. Antioxidants.

[166] Yang L., Yang F., Teng L., Katayama I. (2020). 6-Shogaol protects human melanocytes against oxidative stress through activation of the NRF2-antioxidant response element signaling pathway. Int. J. Mol. Sci..

